# Micro–Nano Water Film Enabled High-Performance Interfacial Solar Evaporation

**DOI:** 10.1007/s40820-023-01191-6

**Published:** 2023-09-22

**Authors:** Zhen Yu, Yuqing Su, Ruonan Gu, Wei Wu, Yangxi Li, Shaoan Cheng

**Affiliations:** State Key Laboratory of Clean Energy Utilization, College of Energy Engineering, Zhejiang University, Hangzhou, 310027 People’s Republic of China

**Keywords:** Micro–nano water film, Interfacial solar evaporation, Solar desalination, Artificial neural networks, PPy sponge

## Abstract

Interfacial solar evaporation holds great promise to address the freshwater shortage. However, most interfacial solar evaporators are always filled with water throughout the evaporation process, thus bringing unavoidable heat loss. Herein, we propose a novel interfacial evaporation structure based on the micro–nano water film, which demonstrates significantly improved evaporation performance, as experimentally verified by polypyrrole- and polydopamine-coated polydimethylsiloxane sponge. The 2D evaporator based on the as-prepared sponge realizes an enhanced evaporation rate of 2.18 kg m^−2^ h^−1^ under 1 sun by fine-tuning the interfacial micro–nano water film. Then, a homemade device with an enhanced condensation function is engineered for outdoor clean water production. Throughout a continuous test for 40 days, this device demonstrates a high water production rate (WPR) of 15.9–19.4 kg kW^−1^ h^−1^ m^−2^. Based on the outdoor outcomes, we further establish a multi-objective model to assess the global WPR. It is predicted that a 1 m^2^ device can produce at most 7.8 kg of clean water per day, which could meet the daily drinking water needs of 3 people. Finally, this technology could greatly alleviate the current water and energy crisis through further large-scale applications.

**Supplementary Information:**

The online version contains supplementary material available at 10.1007/s40820-023-01191-6.

## Highlights

- Micro–nano water film enhanced interfacial solar evaporator enables a high evaporation rate of 2.18 kg m^−2^ h^−1^ under 1 sun.
- An outdoor device with an enhanced condensation design demonstrates a high water production rate of 15.9–19.4 kg kW^−1^ h^−1^ m^−2^.
- A multi-objective predictive model is established to assess outdoor water production performance.

**Supplementary Information:**

The online version contains supplementary material available at 10.1007/s40820-023-01191-6.

## Graphical abstract

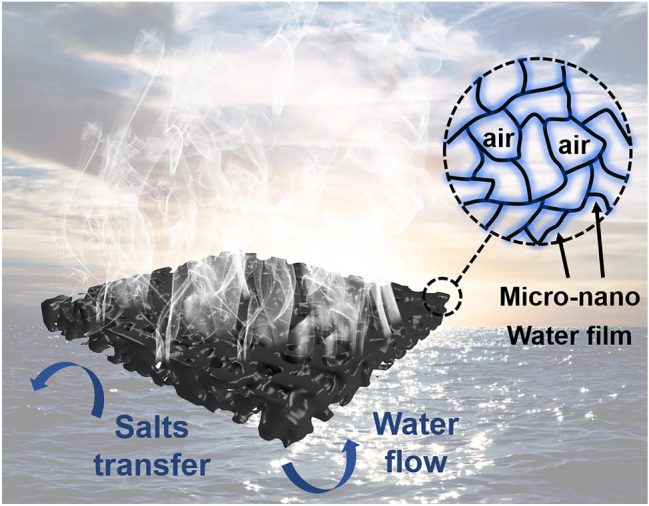

## Introduction

Global water shortage, severe water pollution, growing energy consumption, and global warming have attracted tremendous interest in clean water production in an environmentally friendly manner [1, 2]. Solar-driven seawater desalination technology satisfies the above needs and thus has received increasing attention lately [3, 4]. However, traditional solar stills often suffer from colossal heat loss due to the volume heating way, thereby giving rise to unsatisfactory performance [5, 6]. An emerging solar evaporation technology driven by interfacial heat localization with high energy-conversion efficiency (> 80% under 1 sun) has been developed [7–9]. Recent years have witnessed the significant development of this topic from the aspects of materials design and interfacial engineering [10–12]. The evaporation rate of the interfacial solar evaporators has exceeded the theoretical thermodynamic evaporation limit (1.47–1.61 kg m^−2^ h^−1^ under 1 sun), corresponding to the evaporation efficiency close to 100% [13–18].

Reducing evaporation enthalpy is one of the most effective approaches to achieving a high evaporation rate surpassing the thermodynamic limit [19, 20]. Since the intermediate water in hydrogels possesses a low evaporation enthalpy, hydrogel-based solar evaporators often show a high evaporation rate [21]. Lu’s group proposed a PEDOT/PSS-PVA hydrogel with a fast evaporation rate (2.84 kg m^−2^ h^−1^) [22]. The high evaporation performance benefited from the reduced evaporation enthalpy, enhanced light-harvesting, and strengthened water supply. Similarly, dual network PEDOT/PSS-PAAm hydrogel exhibited a superior evaporation rate of 2.15 kg m^−2^ h^−1^ [23]. Besides the hydrogel, other materials with a tailored porous structure can also attain the same effect [24, 25]. For example, interfacial evaporation is likely enhanced by the microarray and microdroplets, allowing the evaporators a high evaporation rate of 2.16 kg m^−2^ h^−1^ under 1 sun [10]. An interconnected porous photothermal matrix displays a high evaporation performance of 2.2 kg m^−2^ h^−1^ under 1 sun by decreasing the interaction energy of interfacial water molecules [26]. In addition, constructing the 3D evaporation structure is another effective way to enhance the evaporation performance [27, 28]. A nature-inspired low-tortuosity three-dimensional (3D) evaporator achieved a high evaporation rate of 3.0 kg m^−2^ h^−1^ enhanced by environmental energy [29].

It is often overlooked that the pores of the above evaporators are always filled with water throughout the evaporation process, and this excessive water could bring unavoidable heat loss [30]. Therefore, adjusting the water content within the pores to expose more evaporation areas and enhance the heat localization effect could be a promising strategy to improve evaporation performance further [31]. In this context, we propose a novel interfacial solar evaporator based on the micro–nano water film for high-efficiency solar desalination. Porous polypyrrole (PPy)- and polydopamine (PDA)-coated polydimethylsiloxane (PDMS) sponge (denoted as PPy sponge) is prepared here to construct the micro–nano interfacial solar evaporation structure. The as-prepared PPy sponge is employed to investigate how the micro–nano water film governs the evaporation behavior, verified by the evaporation measurements and in situ optical characterization. A homemade device with an enhanced condensation function is then designed for outdoor experiments under the guidelines of computational fluid dynamics (CFD) calculations. The water production performance is continuously recorded with this device over a 40-day test for convincing practical system-level assessment. The cost and performance of the outdoor device are also discussed. Based on the weather conditions and WPR, a multi-objective predictive model is further established through artificial neural networks, which allows us to predict the WPR in different regions and seasons across the globe.

## Experimental Section

### Materials and Regents

Sugar cubes were obtained from Taikoo Sugar Limited (China). PDMS prepolymer and curing agent (Sylgard 184) were purchased from Dow Corning. (3-aminopropyl)triethoxysilane (APTSE) and pyrrole (Py) was purchased from Aladdin. Tris–HCl buffer with a pH of 8.5 were bought from Solarbio. All other chemicals were purchased from Sinopharm Chemical Reagent. All reagents were used without further purification.

### Preparation of the PDMS Sponge

The PDMS sponge was prepared by a sacrificial template method [32, 33]. Typically, sugar cubes were immersed in the mixture of PDMS prepolymer, curing agent, and ethyl acetate (the weight ratio of these contents was 10:1:10). The as-obtained mixture was degassed under vacuum at 25 °C. Then, the sugar cubes were transferred to the oven and cured at 60 °C for 12 h, thereby obtaining sugar cubes with PDMS. The sugar cubes with PDMS were immersed in 90 °C hot water and washed several times. After the sugar cubes dissolution fully, the PDMS sponge was obtained after being washed and dried.

### Preparation of the PDA Sponge

The PDA sponge was prepared by a two-step method [34]. Typically, the as-obtained PDMS sponge was immersed into the 2 wt% APTSE solution with a mixture of ethanol and water (the weight ratio of these contents was 95:5) for 1 h. Then, the PDMS sponge was transferred to the oven and reacted at 110 °C for 30 min. After that, the PDMS sponge was immersed and reacted for 24 h in a mixture of 2 mg mL^−1^ dopamine solution and Tris–HCl buffer with a pH of 8.5. After cleaning with deionized water and drying with Ar flow, the PDA sponge was obtained.

### Preparation of the PPy Sponge

The PPy sponge was prepared by a simple coating method [11, 35]. Typically, 0.345 mL of pyrrole (Py) and 1.12 g of ammonium persulfate (APS) were dissolved into the 50 mL of deionized water, respectively, to form solution A and solution B. The as-obtained PDA sponge was soaked into solution A for 5 min and transferred into a culture dish. And then, an amount of solution B was added to the culture dish and reacted for 30 min at room temperature. Then, PPy sponge was obtained after being taken from the culture dish, washed with deionized water, and dried.

### Characterization

The morphology was observed by the scanning electron microscope (SEM, ZEISS Gemini SEM 300, Germany). The contact angle was calculated by the dynamic contact angle tester (OCA20, Germany). The light absorption was measured by a UV–Vis-NIR spectrophotometer (UV-3101, Japan) equipped with an integrating sphere. The pore size distribution and porosity were measured by AutoPore IV 9600 (Micromeritics Co. Ltd. USA). The changes in surface morphology of the sponge before and after absorbing water were recorded by an optical microscope (Nikon DS-Fi2, Japan). The evaporation enthalpy was obtained by the differential scanning calorimeter (DSC, Mettler Toledo Crop DSC3, Switzerland).

### Indoor Solar Evaporation Experiments

The fabrication method of the evaporator was provided in Supplementary Information. Before the solar evaporation experiment, the evaporator was put on the water container with 100 mL of deionized water for 12 h to achieve water transport balance. The solar evaporation experiment was conducted on a homemade optical system. The evaporation rate was calculated by the mass change of the water container with the evaporator before and after irradiation. The evaporation efficiency of the evaporator was calculated by Eq. (1) where $\dot{m}$ was the evaporation rate (kg m^−2^ h^−1^); $h_{lv}$ was the enthalpy change of the water to vapor (including sensible heat and evaporation enthalpy) (MJ kg^−1^); $q_{{{\text{solar}}}}$ was the solar flux (kW m^−2^) [7, 36].

1 $$\eta = \frac{{\dot{m} \cdot h_{lv} }}{{q_{{{\text{solar}}}} }}$$

The solar desalination experiments were conducted in real seawater (from the East China Sea). The evaporator based on the PPy sponge operated in seawater under 1 sun for 10 h and then transferred to the original seawater for 14 h in the dark, forming a cycle. Unless mentioned, the evaporation performance measurement method in seawater was similar to that in pure water. The water samples, including condensed water and original seawater, were stored for further analysis. The ion concentrations in water samples were measured by an inductively coupled plasma spectrometer (ICP-AES, ICP-6000, UK). The total organic carbon (TOC) was measured by a TOC analyzer (MULTI N/C 3100, Germany). The chemical oxygen demands (COD) were measured according to the manufacturer’s procedure (HACH Method 8000) [37]. The total dissolved solids (TDS) of water samples were measured by an ionic conductivity meter (FG3, Mettler-Toledo, Switzerland).

### Outdoor Experiments

The outdoor experiments were conducted in a homemade device. The outdoor device operated 8 h every day. However, the condensation module and ventilation module inside the device operated 10 min every 1 h (the simulation results show that when the condensation and ventilation module are operated with the optimal parameters, the humidity inside this device can be reduced to about 40% in only 10 min). The weather conditions were recorded in detail every day. Other details were shown in Supplementary Information.

## Results and Discussion

### Design of the Micro–Nano Interfacial Solar Evaporation Structure

**Fig. 1:**
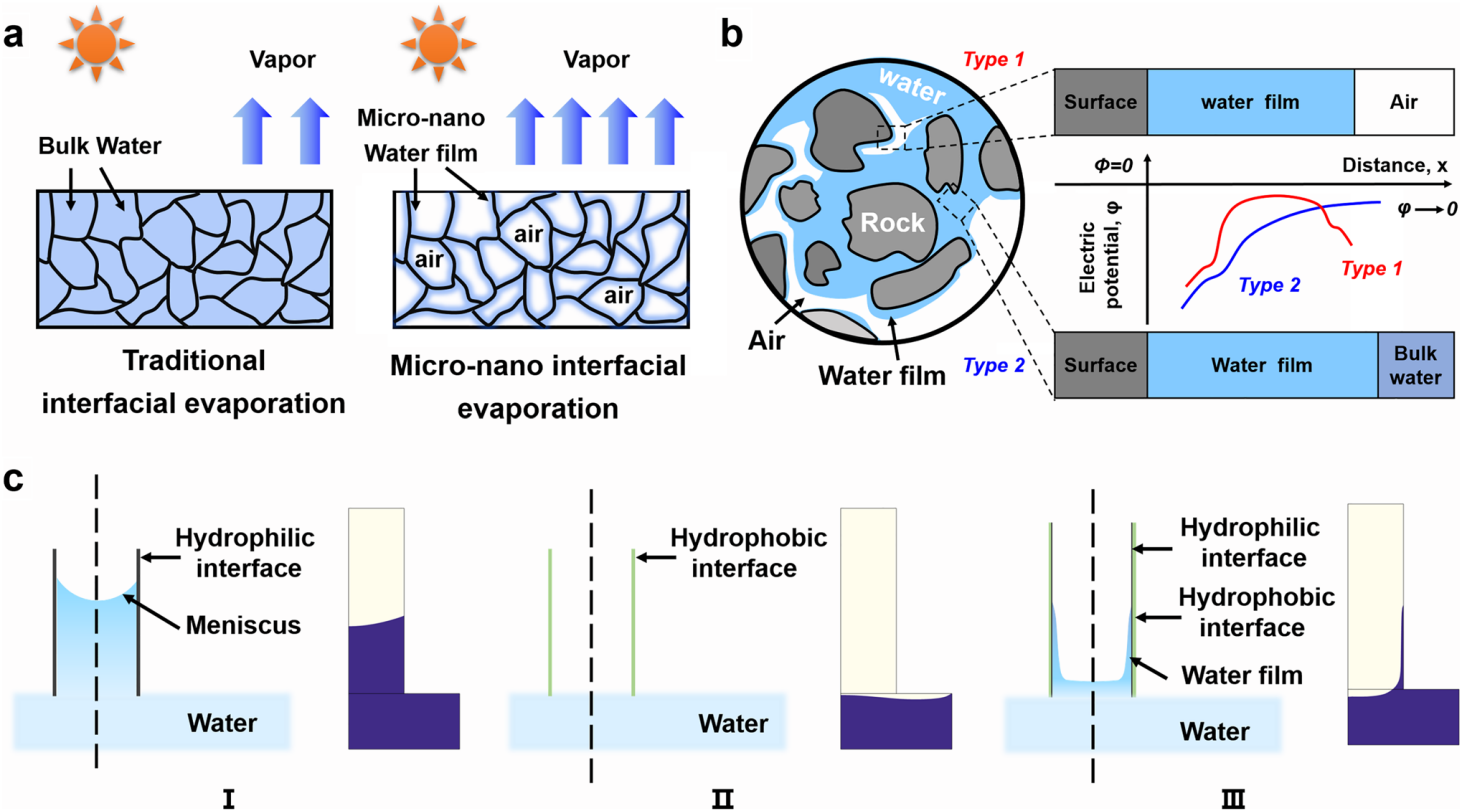
The design of the micro–nano interfacial solar evaporation structure: **a** Schematic of traditional interfacial evaporation structure and micro–nano interfacial evaporation structure; **b** schematic of the existing form of the water in the rock; **c** the schematic and simulated results of the water transfer in the capillary with different interfaces: (I) the hydrophilic interface, (II) the hydrophobic interface, and (III) the hydrophobic interface with a thin hydrophilic layer

For the traditional porous interfacial solar evaporators, the bulk water always fills the whole pores, inevitably escalating parasitic heat loss (Fig. 1a). Suppose water can exist in the form of a micro–nano water film in the porous evaporator (Fig. 1a). In this case, it will enhance heat localization and probably expand the effective evaporation area [38]. This localized design of the water film is named the micro–nano interfacial evaporation structure here. We get the inspiration to design this structure from natural rocks. The water often exists in the rock in two states: one covers the surface of the rock (Type 1 in Fig. 1b), and the other fills the whole pores of the rock (Type 2 in Fig. 1b) [39]. Type 1 in Fig. 1b is very similar to the micro–nano interfacial evaporation structure we are pursuing. In the rock, when the rock has high surface energy, the hydrated ions in the water are distributed on the surface of the rock, so the potential of the water is high at this time. The farther distance from the rock means the lower theoretical potential of the water and finally presents electrical neutrality. When the rock has low surface energy, the hydrated ions in the water will still be distributed on the surface of the rock, but the total amount of hydrated ions adsorbed greatly decreases. At this time, the point away from the surface of the substrate reaches neutrality more rapidly. When it is reduced to a critical value, due to the electric layer effect between the water film and the air, the critical layer between the water film and the air will still absorb a certain number of hydrated ions. The theoretical potential of the water film will also increase with the distance between the air and the water decreasing. Therefore, for rock with low surface energy, the potential of the water first decreases and then increases, and the micro–nano water film could be thus formed on the rock. From this, it can be judged that constructing a structure with low surface energy probably enables to form a micro–nano water film.

To verify above speculation, the rising behavior of water in capillaries with different wettability is simulated. As simulated, the water rises rapidly in the capillary with the hydrophilic interface under capillary force (Figs. 1c-(I) and S1a) while the water not only does not rise but also falls in the capillary with the hydrophobic interface (Figs. 1c-(II) and S1b). As we know, the hydrophobic capillary with low surface energy could not drive the water rise. When a thin hydrophilic layer is attached to the hydrophobic capillary, the whole capillary still exhibits a low surface energy, and the surface energy strengthens as the thickness of the hydrophilic layer increases. It thus can be guessed that water would rise along the capillary wall in the form of the water film instead of the meniscus under the low surface energy. The simulated results have verified this guess (Figs. 1c-(III) and S1c). As mentioned above, attaching a thin hydrophilic layer to the hydrophobic interface may be the critical method for constructing the micro–nano interfacial evaporation structure. We would verify it by the experimental results hereinafter.

### Fabrication and Characterization of Micro–Nano Interfacial Solar Evaporators

**Fig. 2:**
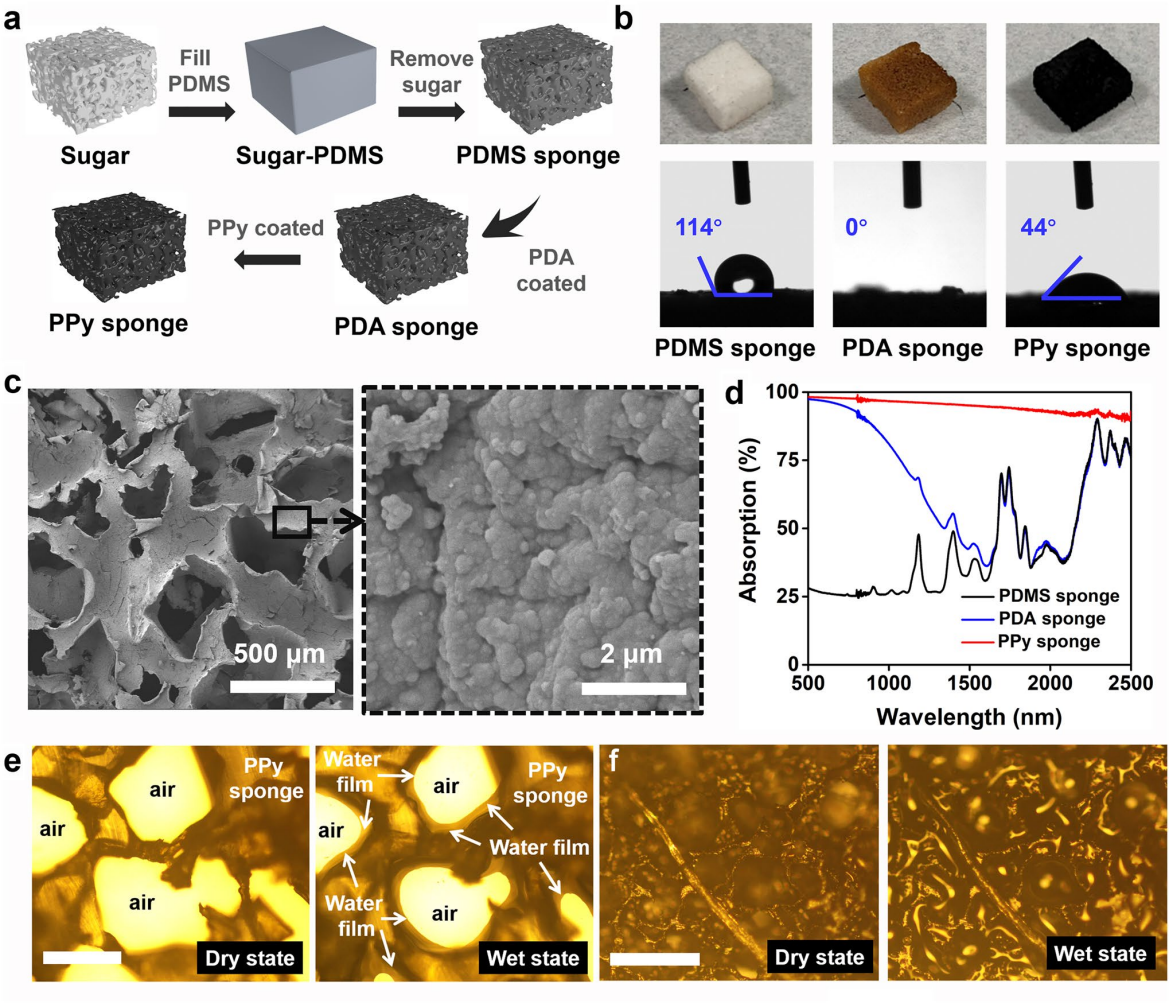
Fabrication and characterization of the PPy sponge. **a** Fabrication process of the PPy sponge; **b** digital photographs and water contact angles of PDMS sponge, PDA sponge, and PPy sponge; **c** SEM images of the PPy sponge; **d** UV–Vis-NIR spectra of the PDMS sponge, PDA sponge, and PPy sponge. The optical micrograph of **e** the PPy sponge and **f** PPy/PDA-PVF sponge. The legend size is 200 µm

We select PDMS as a hydrophobic substrate to construct the micro–nano interfacial evaporation structure because of its durable mechanical properties and remarkable stability in harsh conditions [40]. Porous PDMS sponge could be easily obtained by a simple template method using sugar cubes as sacrificial templates (Fig. 2a). The as-obtained PDMS sponge composes of a macroporous structure (Fig. S2a), and the fiber in the PDMS sponge is smooth (Fig. S2b). We must deposit a thin hydrophilic layer on the PDMS sponge to construct the micro–nano interfacial evaporation structure. PDA coating is selected as the hydrophilic layer, given that PDA coatings can be grown on almost any substrate [41]. However, considering that PDMS substrate has self-healing hydrophobicity, to ensure the stability of the PDA coating, the PDMS sponge was first modified with APTES [42]. Due to the hydrolysis of APTES, a thin film containing –NH_2_ groups is formed on the PDMS sponge. The NH_2_-PDMS sponge was then transferred into the freshly prepared dopamine solution, and the ad-layer of –NH_2_ can be formed on the surface of PDA by Schiff base or Michael addition reaction [43, 44]. Therefore, PDA molecules can be firmly attached to the PDMS sponge through chemical reactions. The detailed reaction mechanism is listed in Fig. S3 [34].

Through the above operations, a PDA-coated PDMS sponge (PDA sponge) is obtained. Compared with the PDMS sponge, the porous structure of the PDA sponge reserves well (Fig. S2c), but many nanoparticles appear on the fibers of the PDA sponge (Fig. S2d). As expected, the PDMS sponge shows hydrophobicity while the PDA sponge presents super hydrophilicity (Fig. 2b). So far, we believe that the PDA sponge has met the basic requirements for forming a micro–nano interfacial evaporator. PDA sponge exhibits brown (Fig. 2b), implying weak light absorption. To further increase light absorption, we deposit a PPy layer in situ on the PDA sponge, thereby obtaining the PPy-coated PDA sponge (PPy sponge). The PPy sponge appears black (Fig. 2b). The hydrophilic structure of the PPy sponge is not destroyed (Fig. 2b, c). Note that the contact angle between the water and PPy sponge increases after PPy deposition, consistent with the previous reports that the hydrophilicity of the substrate decreases after PPy deposition, but the hydrophilic substrate remains hydrophilic [15, 45, 46]. Therefore, it is possible to form a micro–nano water film in the PPy sponge. Many PPy nanoparticles are evenly distributed on the fiber of the PPy sponge (Fig. 2c). These PPy nanoparticles could effectively enhance light absorption and convert the light into thermal energy. Compared to the PDA sponge (~ 70%), the PPy sponge thus enables a high light absorption of ~ 94% (Fig. 2d). Finally, the porosity and pore size distribution of the PPy sponge are recorded in Fig. S4. The results show that the porosity of synthetic PPy sponge is about 70.9%, and most of the pores are in the size range of 100–200 μm.

Then optical characterization is employed to investigate the interfacial change of the PPy sponge before and after wetting. After wetting, the water is attached to the skeleton of the PPy sponge in the form of a micro–nano water film with an average thickness of about 28 μm (Fig. 2e), which fully proves that the PPy sponge we designed belongs to a micro–nano interfacial evaporation structure. We also record other PPy- and PDA-coated hydrophilic sponges before and after wetting (The fabrication method of these sponges is shown in Supplementary Information). Different from PPy sponge, these sponges are filled with water after wetting (Figs. 2f and S5). All in all, it can be concluded that depositing a thin hydrophilic layer on the hydrophobic substrate may be the key to construct the micro–nano interfacial evaporation structure. Note that here we only compare the existing form of water on the hydrophilic substrate and hydrophobic substrate with a hydrophilic layer. As the above simulation and Fig. S6 shown, water cannot enter the hydrophobic sponge through capillary force. Therefore, the existing form of water on the hydrophobic substrate is not studied. In addition, growing the hydrophilic/hydrophobic coating directly on the sugar would probably yield similar results, but the production process is too difficult. We found that the sugar cube would gradually dissolve into the aqueous solution during the fabricated process. It is thus difficult to directly deposit the hydrophilic/hydrophobic coating on the sugar, and the stability of the obtained sponge may be poor. In the future, other stable substrates can be considered to verify the feasibility.

### Indoor Solar Evaporation Experiments

**Fig. 3:**
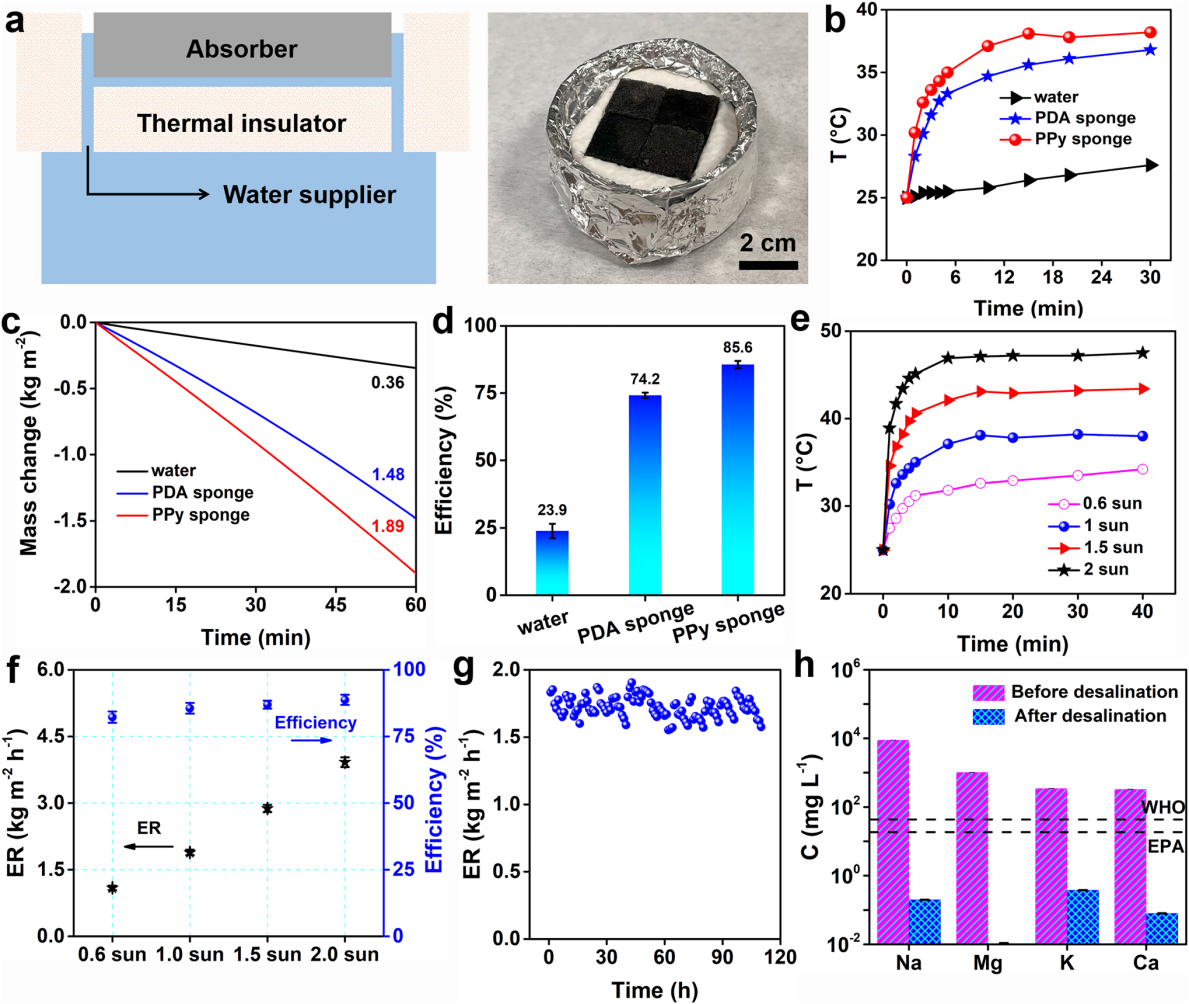
**a** Schematic diagram and digital photographs of the 2D interfacial solar evaporators containing the PPy sponge. The evaporation performance of the evaporator containing the PPy sponge in pure water under 1 sun, taking pure water and PDA sponge as a control: **b** surface temperature curves; **c** mass change curves; **d** evaporation efficiency. The evaporation performance of the evaporator containing PPy sponge in pure water under different solar fluxes: **e** surface temperature curves; **f** evaporation rate and efficiency. **g** long-term evaporation rate of the evaporator based on the PPy sponge in seawater; **h** ion concentrations of seawater before and after desalination

The structure of the solar evaporator containing PPy sponge is shown in Fig. 3a. To avoid extra evaporation, the part of the evaporators not containing the PPy sponge is wrapped. The surface temperature of the dry sponge is traced under 1 sun first. Compared with the PDMS sponge and PDA sponge, the PPy sponge performs a higher temperature rise rate (Fig. S7). Considering the weak water transfer ability of the PDMS sponge (Fig. S6), we only record the surface temperature of the PPy sponge and PDA sponge after absorbing the water under 1 sun. The wet PPy sponge presents a higher temperature rise rate and higher steady-state temperature over pure water and the PDA sponge (Fig. 3b), but the steady-state temperature of the wet PPy sponge (~ 38 °C) is lower than that of the dry PPy sponge (~ 42 °C, Fig. S7).

We then measure the evaporation performance of the evaporator containing the PPy sponge under 1 sun. The evaporator based on the PPy sponge enables a high evaporation rate of 1.89 kg m^−2^ h^−1^, *ca* 5.3 times and 1.3 times those of pure water and PDA sponge, respectively (Fig. 3c). Based on the corrected evaporation enthalpy (Fig. S8), the evaporation efficiency of the evaporator containing PPy sponge is calculated to be 85.6% (Fig. 3d), higher than that of the PDA sponge (74.2%) and pure water (23.9%). The heat loss of the PPy sponge was calculated under 1 sun (The details are provided in Supplementary Note S1). The heat loss of the PPy sponge mainly includes radiation heat loss, convective heat loss, and conduction heat loss, which account for 5.7%, 6.6%, and 1.0% of the total incident energy, respectively. The total heat loss accounts for 13.2% of the total incident energy, and the sum of the heat loss and evaporation efficiency is nearly 100%, implies that the rationality of the evaporation efficiency.

We also evaluate the evaporation performance of the evaporator containing PPy sponge under different solar fluxes. The PPy sponge exhibits a gradually increasing steady-state temperature (Fig. 3e): 34.2 °C (0.6 sun); 38 °C (1 sun); 43.4 °C (1.5 sun) and 47.5 °C (2 sun). Correspondingly, as the solar flux increases from 0.6 sun to 2.0 sun, the evaporation rate accelerates from 1.09 to 3.92 kg m^−2^ h^−1^, and the evaporation efficiency boosts from 82.3 to 88.7% (Fig. 3f). The above results imply PPy sponge enables a satisfactory evaporation performance, even under low solar flux.

Finally, the long-term desalination experiment was conducted in real seawater. The evaporator based on the PPy sponge enables a stable evaporation rate in the seawater under 1 sun (Fig. 3g). Except for the durable mechanical properties (Fig. S9), excellent tolerance in high-salinity brine (Fig. S10), and excellent salt transfer performance (Fig. S11), the stable desalination performance benefits from the intermittent salt discharge mode. In this mode, the salt accumulated in the evaporator under solar irradiation will diffuse back into the bulk seawater under the action of the salinity difference at night, thereby realizing the self-cleaning of the evaporator [47–49]. Therefore, for an evaporator with good salt transfer performance, it is possible to achieve stable desalination only by adjusting the evaporation time and reducing the salinity of the bulk brine [35, 37]. Meanwhile, we measure the salinity of the condensed water produced by the PPy sponge. Compared to the seawater, the salinity of the condensed water is significantly reduced, far lower than the drinking water limit of WHO and EPA (Fig. 3h) [50]. In addition, we also traced the other water quality index of the condensed water. Compared to the seawater, the TOC, COD, and TDS values of the condensed water reduce to ~ 0.8, 2.1, and 3.3 mg L^−1^, respectively (Table S1). The low TOC, COD, and TDS value of the condensed water implies that the PPy sponge possesses outstanding shielding ability against seawater pollutants, and simultaneously the organic polymer of the PPy sponge cannot leak into the condensed water. All the above means that the evaporators based on the PPy sponge possess great potential in seawater desalination.

### Evaporation Mechanism

The PPy sponge, as the concept proof, has verified the superior performance of micro–nano interfacial evaporation structure by solar evaporation experiments. Except for the PPy sponge, the water film in the PDA sponge was also recorded via in situ optical characterization. The thickness of the micro–nano water film in the PDA sponge is significantly greater than that of the PPy sponge (Fig. S12), probably because the PDA sponge is more hydrophilic. However, the PPy sponge exhibits a higher evaporation rate and a lower evaporation enthalpy, and it thus can be concluded that the micro–nano water film affects the evaporation performance. To further demonstrate it, an in situ water supply-evaporation experiment was conducted in a homemade system (Fig. S13) under 1 sun. Before the water supply-evaporation experiment, the PPy sponge naturally absorbs water by the capillary force. In the experiment, the water supply rate of the PPy sponge is controlled by the micro syringe pump. The optical characterization is employed to observe the water film change in the PPy sponge.

**Fig. 4:**
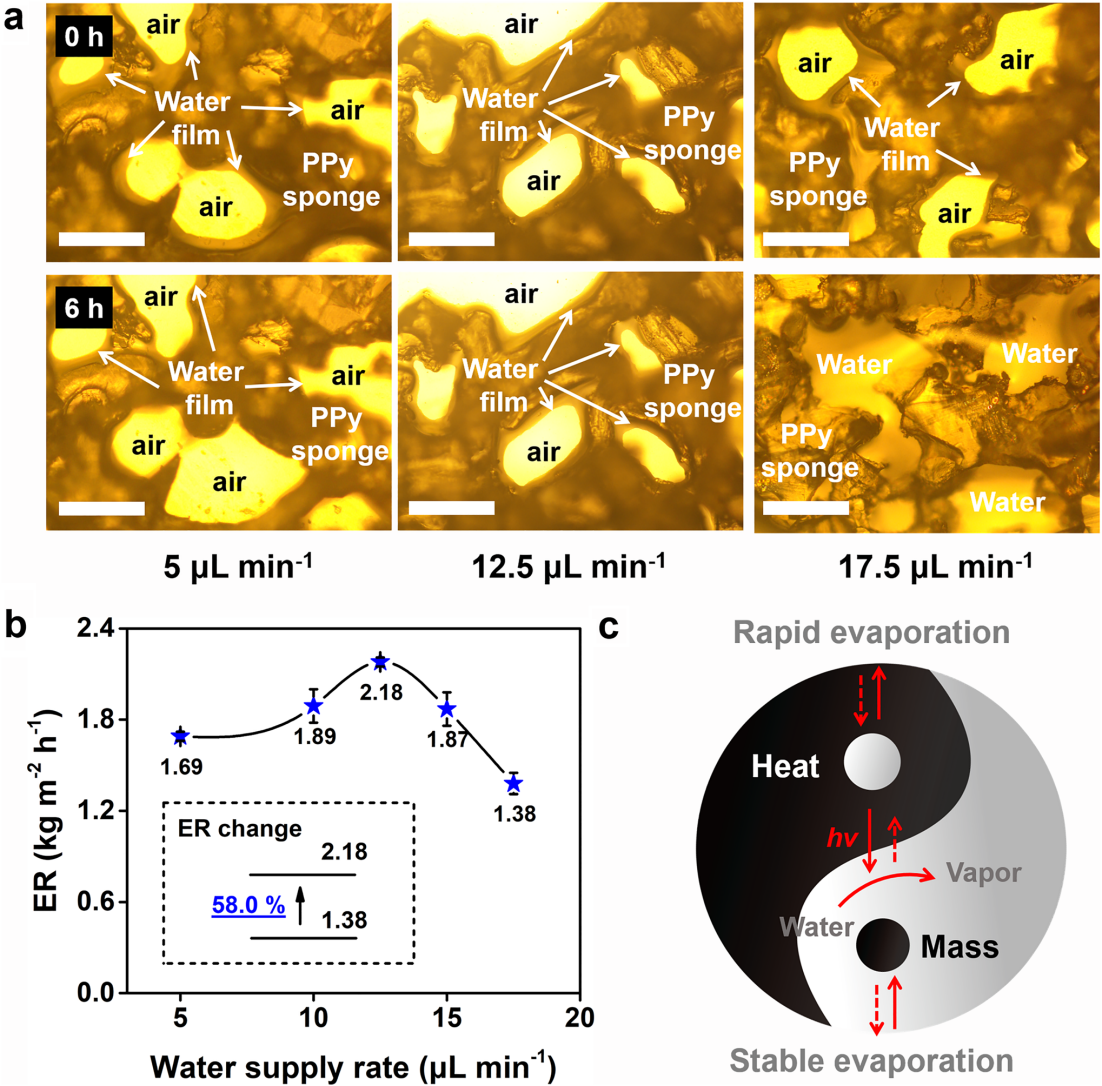
**a** Evaporation rate of the PPy sponge with the different water supply rate; **b** optical micrograph of the PPy sponge under the different water supply rate. The legend size is 200 µm. **c** schematic for heat and mass transfer balance

Under the 5 μL min^−1^ of the water supply rate, the water film on the PPy sponge gradually thins with evaporation (Fig. 4a), implying a low water supply rate. As expected, the PPy sponge presents a low evaporation rate of 1.69 kg m^−2^ h^−1^ at this time (Fig. 4b). When the water supply rate increased from 5 to 12.5 μL min^−1^, the thickness of the water layer changes little before and after evaporation experiments (Fig. 4a), implying the evaporation rate and water supply rate reach equilibrium. In this case, the PPy sponge exhibits a high evaporation rate of 2.18 kg m^−2^ h^−1^ (Fig. 4b). When the water supply rate further increased to 17.5 μL min^−1^, the pores of the PPy sponge are filled with water thoroughly. In this case, the PPy sponge belongs to the traditional interfacial evaporation structure instead of the micro–nano interfacial evaporation structure. As expected, the evaporation rate of the PPy sponge (1.38 kg m^−2^ h^−1^, Fig. 4b) is similar to that of the traditional interfacial evaporator (such as PPy/PDA-ME sponge, PPy/PDA-PVF sponge, and PPy/PDA-PU sponge) (Table S2). Overall, the suitable water film in the micro–nano interfacial evaporation structure is the key to maintaining the high evaporation rate.

In the above experiments, we want to convey a design principle of the high-efficiency interfacial solar evaporator, that is, “heat and mass transfer balance” (Fig. 4c). The too fast mass transfer rate (water transfer rate) will increase the parasitic heat loss, and the too slow mass transfer rate will limit the evaporation kinetics, which both leads to low evaporation performance. Therefore, it is necessary to balance heat and mass transfer rates to achieve stable and efficient solar evaporation. By adjusting the water transfer rate, the evaporation rate of the PPy sponge can be further enhanced from 1.89 to 2.18 kg m^−2^ h^−1^ under 1 sun, already higher than many 2D reported evaporators (Fig. S14).

3D micro–nano interfacial structure seems to have a higher evaporation rate [51, 52]. Taking PPy sponge as an example, with the height of PPy sponge increasing from 0.8 to 3.2 cm, the evaporation rate increasing from 3.1 to 5.9 kg m^−2^ h^−1^ (Fig. S15). Considering the larger evaporation area, we only use the 2D interfacial evaporation structure to highlight the excellent evaporation performance of the micro–nano interfacial evaporation structure here. As for 3D micro–nano interfacial evaporation structure, it possesses a higher performance but needs further study. Finally, we would like to point out that the sugar was used as the template to construct a micro–nano interfacial solar evaporation structure. Considering that the sugar cube would dissolve in the aqueous solution, the sugar was removed in advance. To avoid generating organic wastewater, some stable substrates, such as metal foam, can be used to construct micro–nano interfacial evaporation structures in the future.

### Outdoor Solar Desalination

**Fig. 5:**
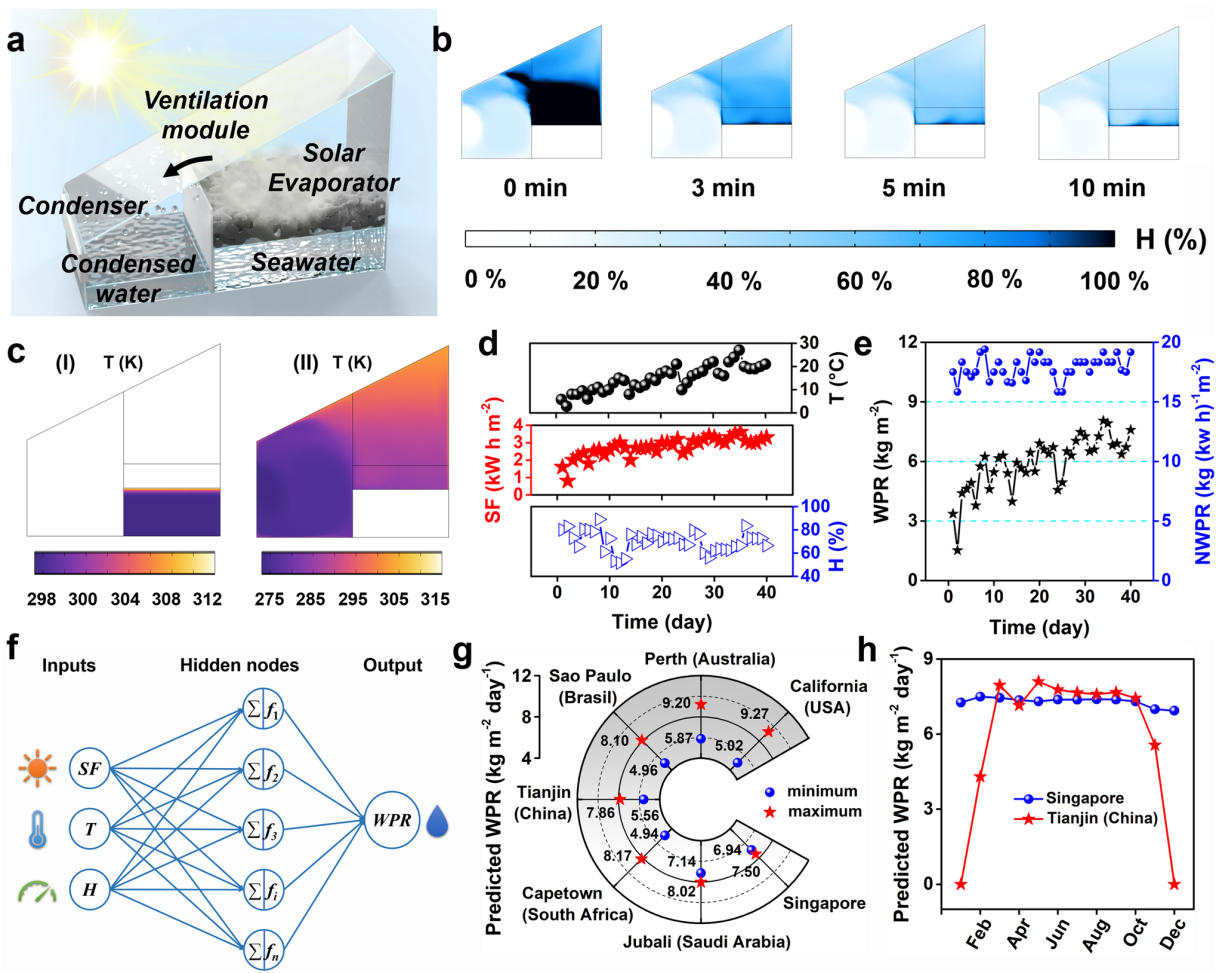
Measurements and prediction of outdoor water production performance: **a** schematic diagram of the homemade device in outdoor experiments; **b** humidity field of the homemade device under the optimized operated parameters simulated by COMSOL software; **c** temperature field of (I) the evaporator and (II) the device simulated by COMSOL software; **d** daily total solar flux (SF), temperature (T), and humidity (H) in outdoor experiments; **e** water production rate (WPR) and WPR normalized based on daily total solar flux (NWPR) in outdoor experiments; **f** the prediction process of outdoor WPR; **g** predicted outdoor WPR in 7 water-scarce regions all over the world; **h** predicted outdoor WPR of Singapore and Tianjin (China) in every month

To achieve high-efficiency solar desalination, the outdoor device for interfacial solar evaporators should generally satisfy at least two terms: rapid water evaporation and efficient vapor condensation [53, 54]. The reported traditional outdoor devices often achieve vapor condensation relying on the single slope cover, which presents a poor condensed efficiency owing to the low thermal conductivity of the air. In addition, the humidity inside these devices becomes higher and higher with water evaporation, thereby inhibiting the evaporation process. The water condensed on the top of the device will hinder light absorption, also reducing the WPR. Based on the above three reasons, these traditional devices usually suffer from unsatisfactory outdoor performance. To address these issues, we have introduced condensation and ventilation modules to the traditional one (Fig. 5a). The ventilation module provides forced convection conditions and accelerates the mass transfer process between the two chambers. The vapor generated in the evaporation chamber is quickly transferred to the condensation chamber by the ventilation module for condensation. The condensation module achieves the condensation process. Through the coupling of the condensation module and the ventilation module, three issues of the traditional device can be addressed well in theory. We believe that our device containing a high-efficiency interfacial solar evaporator should have an outstanding WPR. In the following, we will further examine and determine it.

The COMSOL software is employed to optimize the operating parameters of the homemade device (The simulation details are provided in Supplementary Note S2). The humidity reflects the condensed effect, in which the lower humidity always means a better-condensed performance. The condenser is located inside the condensation chamber. When the condensing temperature is 5 °C, and the ventilation rate is 1 m s^−1^, the humidity above the evaporation chamber is close to 100% (Fig. S16). When the ventilation rate increases to 3 m s^−1^, the humidity of the evaporation chamber drops to ~ 40% in 10 min. At this time, further increasing the ventilation rate cannot significantly reduce the humidity of the evaporation chamber. Considering energy consumption, we believe 3 m s^−1^ is the optimized ventilation rate. The condensing temperature is optimized when the ventilation rate is set as 3 m s^−1^, and the results are shown in Fig. S17. When the condensing temperature is − 5 °C, the overall humidity of the condensation chamber is lower than 40%, and as the condensing temperature gradually increases. When the condensing temperature rises to 5 °C, the humidity of the condensation chamber is still low. At this time, further increasing the condensing temperature cannot significantly reduce the humidity of the evaporation chamber. Therefore, we believe that 5 °C is the optimized condensing temperature of the condenser. Note that the above optimum parameters are also available for different initial humidity conditions (Fig. S18).

When operating this device with optimum operating parameters, the condenser ensures that the interior of the condensation chamber has a lower temperature, thereby achieving efficient vapor condensation. At this time, the humidity inside the device drops rapidly (Fig. 5b). Meanwhile, the temperature of bulk seawater is about 25 °C while the evaporator shows a high temperature of 40 °C (Fig. 5c-(I)), which could be attributed to the outstanding heat localization ability. The condensation chamber shows a low average temperature (~ 5 °C, Fig. 5c-(II)), and the vapor could be rapidly condensed. In comparison, the temperature of the evaporation chamber is still very high (~ 35 °C, Fig. 5c-(II)), which can ensure effective evaporation.

The outdoor device with an area of 0.32 m^2^ is constructed based on the simulated results, as shown in Fig. S19, and the detailed size of the homemade device is provided in Fig. S20. The large-size PPy sponge with an area of 0.12 m^2^ is used as the evaporator here (Fig. S21). First, we measure the outdoor WPR in different operating modes to verify the enhanced condensation effect. When neither the ventilation fan nor the condenser is on, this device is similar to the traditional outdoor device for the interfacial solar evaporator. In 2 days, the device exhibits a low WPR of 1.7–2.8 kg m^−2^ (Table S3). When only the condenser is on while the ventilation fan is off, the WPR increases little. Considering the relatively large mass transfer distance between the evaporation and the condensation chamber, the vapor generated by the evaporator cannot quickly diffuse into the condensation chamber to be condensed without the ventilation module, so the WPR is still low. When only the ventilation fan is on while the condenser is off, the WPR further increases to 3.2–3.9 kg m^−2^, which is consistent with the reported work that forced convection conditions could present a high evaporation performance [55]. When both the ventilation fan and the condenser are on, this device exhibits a high WPR of 5.1–6.8 kg m^−2^. The evaporation area of the absorber is 0.12 m^2^, and the area of the solar panel is about 0.128 m^2^. The water production rate of the device only increases to 3.51–5.79 kg m^−2^ if the corresponding area of the solar panel would be utilized as an additional evaporative surface, which is still lower than our design (5.1–6.8 kg m^−2^). Therefore, it can be concluded that under the combined effect of the condenser and the ventilation fan, the WPR can be significantly improved. Note that under the similar outdoor weather conditions (Table S4), the WPR of the device containing the PPy sponge is also higher than that of the other PPy/PDA-coated hydrophilic sponge (Table S5), which implies the superior performance of the micro–nano interfacial evaporation structure.

Then we measure the WPR of this device. In 40 days, the outdoor temperature rises with the daily total solar flux increasing, while the outdoor humidity exhibits little association with solar flux (Fig. 5d). Correspondingly, the daily total WPR of this device escalates from 1.52 to 8.05 kg m^−2^ within 40 days (Fig. 5e). The WPR of this device fluctuated greatly within 40 days, probably caused by the widely different weather conditions, especially the light conditions. In the 40 days, the maximum and minimum daily total solar flux are 0.8 and 3.6 kW h m^−2^. To better compare the outdoor water production performance of the devices under different total solar flux, we normalize daily total WPR (unit: kg m^−2^) based on the daily total solar flux (unit: kW h m^−2^) and the area of the PPy sponge (m^2^). Then, a new index is obtained, that is, WPR normalized based on daily total solar flux (NWPR; unit: kg kW^−1^ h^−1^ m^−2^). As calculated, this device creates a high NWPR of 15.9–19.4 kg kW^−1^ h^−1^ m^−2^.

This device does not present the highest WPR when the daily solar flux exhibits maximum, which implies that the WPR should be related to many factors, such as solar flux, temperature, and humidity. To better predict the WPR, we establish a model based on the above parameters by artificial neural networks (Fig. 5f). This model enables an excellent fitting effect when predicting the WPR (Fig. S22). To evaluate the potential for alleviating the freshwater crisis, we predict the WPR of the device in 7 water-scarce regions worldwide (The details are provided in Supplementary Note S3). As predicted, this device with an evaporation area of 1 m^2^ could produce 2.7–7.8 kg of water daily (Fig. 5g). In addition, we predict the WPR of this device in different seasons (Figs. 5h and S23). For example, this device enables a stable average WPR of 7.3 kg m^−2^ in Singapore annually. In comparison, this device exhibits a stable WPR of 7.7 kg m^−2^ in Tianjin (China) only from March to October while presenting a low WPR from November to February of the next year, which could be attributed to the freezing period outdoors. Overall, the device shows satisfactory water production performance in the non-freezing seasons. For some areas with water and electricity shortages, the device can perhaps significantly alleviate the plight of local water shortages [56].

## Conclusion

We propose a novel interfacial evaporation structure based on the micro–nano water film, which demonstrates significantly improved evaporation performance, as experimentally verified by PPy- and PDA-coated PDMS sponge (denoted as PPy sponge). The simulation tools are employed to guide the designing process. 2D evaporator based on the PPy sponge realizes an enhanced evaporation rate of 2.18 kg m^−2^ h^−1^ under 1 sun by fine-tuning the interfacial water film. The optical characterization reveals that the micro–nano interfacial evaporation structure enables a better evaporation performance than the traditional one. Then, a homemade device containing a micro–nano interfacial solar evaporator and an enhanced condensation design is employed for outdoor experiments based on the simulated results. In the 40 days, this device creates a high water production rate of 15.9–19.4 kg kW^−1^ h^−1^ m^−2^. In addition, we establish a multi-objective model to predict the water production rate by artificial neural networks based on outdoor experiments. As predicted, this device with an evaporation area of 1 m^2^ could produce 7.8 kg of water daily, which could meet 3 peoples’ daily drinking water needs. In conclusion, this work provides a novel way for efficient, clean water production and gives new sights to large-scale interfacial solar desalination.

### Supplementary Information

Below is the link to the electronic supplementary material.

Supplementary file1 (PDF 2678 kb)
