# Supplementary material for: Micro–Nano Water Film Enabled High-Performance Interfacial Solar Evaporation
Source: Nanomicro Lett. 2023 Sep 22;15:214. doi: 10.1007/s40820-023-01191-6 (PMC10516847; doi:10.1007/s40820-023-01191-6)
Supplement: Supplementary file 1 — Supplementary file1 (PDF 2678 kb) [file 40820_2023_1191_MOESM1_ESM.pdf]

Supporting Informaiton for

Micro-Nano Water Film Enabled High-Performance Interfacial Solar Evaporation

Zhen Yu[1], Yuqing Su[1], Ruonan Gu[1], Wei Wu[1], Yangxi Li[1], Shaoan Cheng[1, *]

[1]State Key Laboratory of Clean Energy Utilization, College of Energy Engineering, Zhejiang University, Hangzhou 310027, P. R. China

[*]Corresponding author. E-mail: shaoancheng@zju.edu.cn (Shaoan Cheng)

S1 Supplementary Method

S1.1 Fabrication of Other PDA Coated Hydrophilic Sponge

The preparation process of other PDA-coated hydrophilic sponges is similar to that of the PDA sponge, except that the substrates of these sponges are melamine sponges, polyvinyl formal sponges, and polyurethane sponges, respectively. PDA-coated melamine sponge, PDA-coated polyvinyl formal sponge, and PDA-coated polyurethane sponge are abbreviated as PDA-ME sponge, PDA-PVF sponge, and PDA-PU sponge, respectively.

S1.2 Fabrication of Other PPy and PDA Coated Hydrophilic Sponge

The preparation process of other PPy and PDA-coated hydrophilic sponges is similar to that of the PPy sponge, except that the substrates of these sponges are PDA-ME sponge, PDA-PVF sponge, and PDA-PU sponge, respectively. PPy and PDA-coated melamine sponge, PPy and PDA-coated polyvinyl formal sponge, PPy and and PDA-coated polyurethane sponge are abbreviated as PPy/PDA-ME sponge, PPy/PDA-PVF sponge, and PPy/PDA-PU sponge, respectively.

S1.3 Fabrication Method of the Interfacial Solar Evaporators

The structure of the solar evaporator containing the PPy sponge was shown in **Fig. 3a**. The evaporator consisted of thermal insulation, water channel, absorber, and shield. A thermal insulation foam with a size of 3.6 cm×3.6 cm×0.8 cm was used as the support and insulator. Non-woven fabric with a length of 16 cm and a width of 3.6 cm was laid on the thermal insulation foam as the two-dimensional water channel. The absorber with an area of 3.6 cm×3.6 cm was placed on the non-woven fabric. To avoid the sidewall evaporation of the absorber, the absorber was embedded in the shield (the inside diameter: 3.6 cm and the outer diameter: 6 cm).

S1.4 In-situ Water Supply and Evaporation Experiments

Before the solar evaporation, the PPy sponge was put on the traditional interfacial solar evaporator for 12 h to achieve the water transfer balance. The wet PPy sponge was placed in a cavity constituted of thermal insulation foam. Only the top of the PPy sponge was exposed to the air. The water supply to the PPy sponge in the solar evaporation experiments was controlled by a micro syringe pump (Longer, LSP01-3A, China). The mass change of the wet PPy sponge and cavity after solar irradiation was used to calculate the evaporation rate. The changes in surface morphology of the PPy sponge were recorded by an optical microscope (Nikon DS-Fi2, Japan).

S1.5 Outdoor Experiments

The ventilation fan was purchased from Xingyao Solar Technology (Dongguan, Guangdong, China), with a maximum working voltage of 12 V and a maximum working current of 2 A. The condenser was purchased from Yuliang Electronic Technology (Huizhou, Guangdong, China), with a maximum working voltage of DC 12 V and a maximum working current of 0.6 A. The ventilation speed and condensing temperature can be adjusted according to the needs of users. The whole device was powered by a solar panel that was purchased from Sikade Technology (Nanjing, Jiangsu, China). The long-term outdoor experiments were conducted in Hangzhou (China) for 160 days and 40 days were selected to analyze. Before long-term outdoor experiments, we first compared the water production rate of this device in different operation mode, taking days with similar weather conditions for analysis. The operating parameters were same as the long-term outdoor experiments. The weather conditions were shown in **Table S6**.

S2 Note S1: Heat Loss Calculation

The absorbed solar energy by the evaporator Q_{solar} was calculated by **Eq. (S1)** where A was the area of the absorber, and q_{solar} was the total energy of the incident light.

$$Q_{solar} = Aq_{solar} \tag{S1}$$

The heat loss was mainly composed of radiation loss, convection loss and conduction loss.

The radiation loss Q_{rad} was calculated by **Eq. (S2)** where A_{eff} was the area of the absorber (3.5×3.5 cm), ε was the emissivity (here about 0.78), and σ was the Boltzmann's constant (5.67×10^{-8} W m^{-2} K^{-4}), T_1 was the steady-state average temperature of the absorber (K), and T_a was the ambient temperature (K).

$$Q_{rad} = A_{eff}\varepsilon\sigma(T_1^4 - T_0^4) \quad \text{(S2)}$$

The convective loss Q_{conv} was calculated by **Eq. (S3)** where A_{eff} was the area of the absorber, h_{eff} was the convective heat transfer coefficient (5 W m^{-2} K^{-1}), T_1 was the steady-state average temperature of the absorber (K), and T_a was the ambient temperature (K).

$$Q_{conv} = A_{eff}\, h_{eff}\,(T_1 - T_0) \quad \text{(S3)}$$

The conduction loss Q_{cond} was calculated by **Eq. (S4)** where C is the specific heat capacity of water (4.2 J g^{-1} K^{-1}), m is the mass of water in the bottom tank (g), $\triangle T$ was the temperature change of water in the bottom tank (K) within a certain irradiation time.

$$Q_{cond} = Cm\triangle T \quad \text{(S4)}$$

The temperature parameters we used to calculate the heat loss were as follows:

T_1 (K)	T_a (K)	$\triangle T$ (K)
311.1	299.8	1.1

The heat loss normalized based on the solar flux was as follows:

Q_{rad}	Q_{conv}	Q_{cond}	Total
5.7 %	6.6 %	1.0 %	13.2 %

S3 Note S2: COMSOL Simulation

S3.1 COMSOL Simulation for Capillary Filling in the Different Interface

A transient-state model was established to study the capillary filling behavior in the different interfaces. The Level Set Method and Phase Field Method were used here [S1]. The capillary can be simplified as a 2D model as follows:

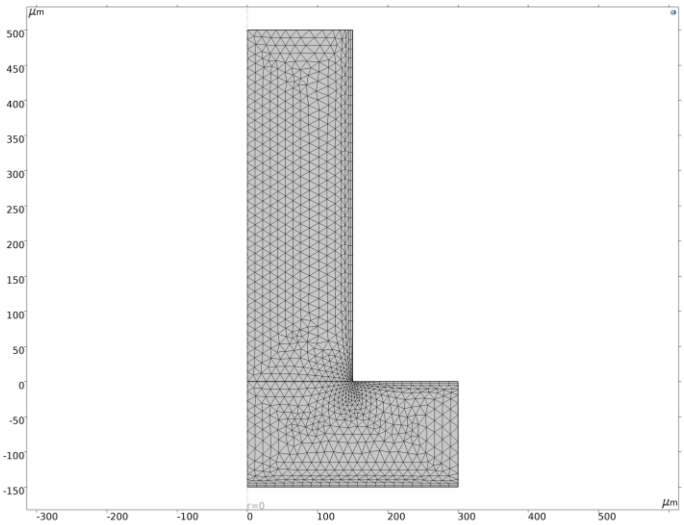

The Level Set interface automatically set up the equations for the convection of the interface. The transport of the fluid interface separating the two phases was given by **Eq. (S5):**

$$\frac{\partial\varphi}{\partial t}+\boldsymbol{u}\cdot\nabla\varphi=\gamma\nabla\cdot\left(\varepsilon\nabla\varphi-\varphi(1-\varphi)\frac{\nabla\varphi}{|\nabla\varphi|}\right) \tag{S5}$$

where φ defined 0 in the air and defined 1 in the water; $\boldsymbol{u}$ was velocity field in fluid; γ was the mobility; ε was the interface thickness parameter.

In the Phase Field interface, the two-phase flow dynamics was governed by a Cahn-Hilliard equation. The equation tracked a diffuse interface separating the immiscible phases. The diffuse interface was defined as the region where the dimensionless phase field variable went from -1 to 1. When solved in COMSOL Multiphysics, the Cahn-Hilliard equation was split up into two equations:

$$\frac{\partial\varphi}{\partial t}+\boldsymbol{u}\cdot\nabla\varphi=\nabla\frac{\gamma^{\lambda}}{\varepsilon^2}\nabla\psi \tag{S6}$$

$$\psi=-\nabla\cdot\varepsilon^2\nabla\varphi+(\varphi^2-1)\varphi \tag{S7}$$

where λ was the mixing energy density. The Navier–Stokes equations described the transport of mass and momentum for fluids of constant density. In order to account for capillary effects, it was crucial to include surface tension in the model. The Navier–Stokes equations were then:

$$\rho\frac{\partial\boldsymbol{u}}{\partial t}+\rho(\boldsymbol{u}\cdot\nabla)\boldsymbol{u}=\nabla\cdot[-p\cdot\boldsymbol{I}+\mu(\nabla\boldsymbol{u}+(\nabla\boldsymbol{u})^T)]+\boldsymbol{F}+\rho g \tag{S8}$$

$$\nabla\cdot\boldsymbol{u}=0 \tag{S9}$$

where ρ was density of fluid; p was the pressure of fluid; $\boldsymbol{I}$ was turbulence intensity; μ was dynamic viscosity; $\boldsymbol{F}$ was external force acting on the fluid.

The capillary filling in the different interface was controlled by the different surface tension and water contact angle.

S3.2 COMSOL Simulation When Designing the Outdoor Device

A transient-state model was established to study the temperature field and humidity field of the outdoor device by the commercial software COMSOL Multiphysics [S2].

The outdoor device can be simplified as a 2D model as follows:

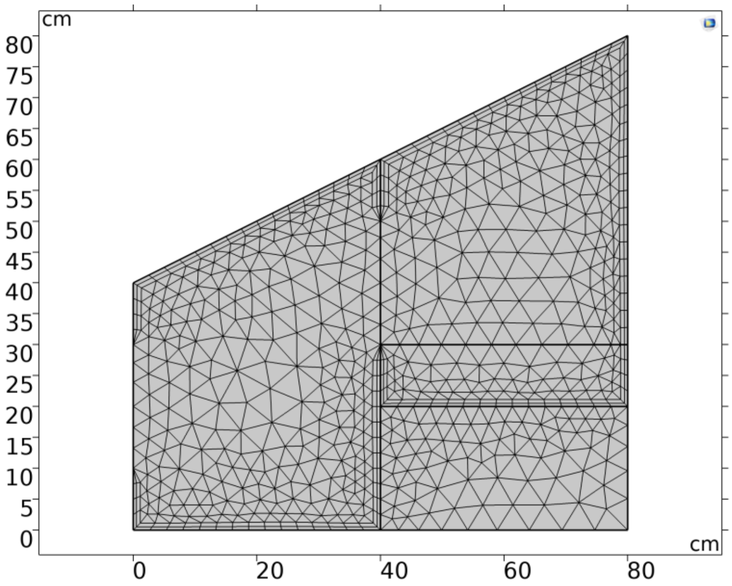

Vapor flow in outdoor device was governed by turbulence equations where ρ was density of fluid; $\boldsymbol{u}$ was velocity field in fluid; p was the pressure of fluid; $\boldsymbol{I}$ was turbulence intensity; μ_T was turbulent viscosity coefficient; μ was dynamic viscosity; T was the temperature of fluid; $\boldsymbol{F}$ was external force acting on the fluid.

$$\rho(\boldsymbol{u}\cdot\nabla)\boldsymbol{u}=\nabla\cdot[-p\cdot\boldsymbol{I}+(\mu_T+\mu)(\nabla\boldsymbol{u}+(\nabla\boldsymbol{u})^T)]+\boldsymbol{F} \quad \text{(S10)}$$

$$\rho\nabla\cdot\boldsymbol{u}=0 \quad \text{(S11)}$$

Turbulent viscosity coefficient (μ_T) was calculated by **Eq. S12** where C_μ was model coefficients; k was the turbulence kinetic energy; σ_ε was the Prandtl (Pr) number corresponding to the turbulent dissipation ε.

$$\mu_T=\rho C_\mu\frac{k^2}{\sigma_\varepsilon} \quad \text{(S12)}$$

Heat transfer process in outdoor device was determined by the thermal equation where ρ was the density; C_p was the specific heat capacity; T was the temperature; t was time; k was the thermal conductivity; $\boldsymbol{q}$ was the heat flux. The properties of the working fluid were derived from the COMSOL material library.

$$\rho C_p \frac{\partial T}{\partial t} + \rho C_p \boldsymbol{u} \cdot \nabla T + \nabla \cdot \boldsymbol{q} = 0 \tag{S13}$$

$$\boldsymbol{q} = -k \nabla T \tag{S14}$$

Humidity change process in outdoor device was described by the **Eq. (S15)** where $w(\varphi_w)$ was the humidity (%); ρ_g and ρ_l were the density of the vapor and water (kg m^{-3}); $\boldsymbol{u_g}$ and $\boldsymbol{u_l}$ were the velocity of the vapor and water (m s^{-1}) w_v was the mass fraction of vapor (%); D was the diffusion coefficient (m^2 s^{-1}).

$$\frac{\partial w(\varphi_w)}{\partial t} + \rho_g \boldsymbol{u_g} \cdot \nabla w_v + \nabla \cdot \boldsymbol{g_w} + \boldsymbol{u_l} \cdot \nabla \rho_l = 0 \tag{S15}$$

$$\boldsymbol{g_w} = -\rho_g D \nabla w_v \tag{S16}$$

Before establishing the governing equation of the condenser, we made the following assumptions: 1) Humid air was a Newtonian fluid composed of dry air and water vapor. The physical parameters of the fluid were constant. 2) The contact thermal resistance of the liquid film and the condenser plate was neglected. The influence of ambient thermal radiation and viscous dissipation was also neglected.

The governing equation for the condensation side of the condenser was shown as follows:

1. Continuity equation:

$$\frac{\partial \rho_a \alpha_a}{\partial t} + \nabla(\rho_a \alpha_a \boldsymbol{u}) + S_m = 0 \tag{S17}$$

$$\frac{\partial \rho_w \alpha_w}{\partial t} + \nabla(\rho_w \alpha_w \boldsymbol{u}) - S_m = 0 \tag{S18}$$

$$\alpha_a + \alpha_w = 1 \tag{S19}$$

Where α_a and α_w were the volume fraction of humid air and condensed water; ρ_a and ρ_w were the density of humid air and condensed water; $\boldsymbol{u}$ was the velocity; S_m

was the quality source term.

2. Momentum Conservation Equation:

$$\frac{\partial(\rho \boldsymbol{u})}{\partial t}+\nabla(\rho \boldsymbol{u})+\nabla p-\mu\nabla^2\boldsymbol{u}-G-F_\sigma=0 \tag{S20}$$

$$F_\sigma=\sigma\frac{2\rho\kappa_w\nabla\alpha_w}{(\rho_a+\rho_w)} \tag{S21}$$

Where p was the static pressure; ρ and μ were the density and dynamic viscosity; G was momentum source term; σ was surface tension; κ_w was the curvature of the gas-liquid interface.

3. Energy conservation equation:

$$\frac{\partial(\rho h)}{\partial t}+\nabla(\rho \boldsymbol{u} h)-\nabla(\frac{\lambda}{C_p}\nabla T)-S_T=0 \tag{S22}$$

Where h was the fluid specific enthalpy; λ was the thermal conductivity; C_p was specific heat capacity; S_T was the energy source term.

The composition change at the condensing side was described by **Eq. (S23)** and **Eq. (S24)** where W_a^v and W_a^a were the mass fraction of dry air and water vapor in humid air.

$$\frac{\partial(\rho_a\alpha_a W_a^v)}{\partial t}+\nabla(\rho_a\alpha_a W_a^v\boldsymbol{u})+\nabla\alpha_a\boldsymbol{J}_{\boldsymbol{a}}^{\boldsymbol{v}}-S_s=0 \tag{S23}$$

$$W_a^a+W_a^v=1 \tag{S24}$$

S4 Note S3: Prediction for Outdoor Water Production Performance

The prediction of outdoor water production performance was conducted by the artificial neural network in ***MATLAB 2019a***. The temperature, solar flux, humidity, and water production rate recorded in 40 days were used as the input variables, while the predicted water production rate was the output variable. To determine the appropriate number of neurons in the hidden layer, 10 ~ 20 neuron were loaded for modeling. After optimizing through the Bayesian regularization algorithm, the best number of neurons was obtained based on the R^2. For all modeling, 85% of the data was used for training, 5% for validation, and the remaining 10% for additional testing. All modeling processes were repeated 5 times to improve the model's prediction performance and accuracy. The

original codes were shown below**.** According to the water scarcity index, 8 regions or cities with high water scarcity were selected for prediction. The local total solar flux and average temperature were used as input variables when predicting the water production performance of different regions or cities worldwide. The specific parameters were provided in **Table S7**.

S5 Original Code of the Artificial Neural Network

```
% Solve an Input-Output Fitting problem with a Neural Network

% Script generated by Neural Fitting app

% Created 18-Jul-2022 11:08:12

%

% This script assumes these variables are defined:

%

%     x - input data.

%     y - target data.

x = x';

t = y';

% Choose a Training Function

% For a list of all training functions type: help nntrain

% 'trainlm' is usually fastest.

% 'trainbr' takes longer but may be better for challenging problems.

% 'trainscg' uses less memory. Suitable in low memory situations.

trainFcn = 'trainlm';    % Levenberg-Marquardt backpropagation.

% Create a Fitting Network

hiddenLayerSize = 10;

net = fitnet(hiddenLayerSize,trainFcn);

% Choose Input and Output Pre/Post-Processing Functions

% For a list of all processing functions type: help nnprocess

net.input.processFcns = {'removeconstantrows','mapminmax'};

net.output.processFcns = {'removeconstantrows','mapminmax'};

% Setup Division of Data for Training, Validation, Testing
```

```
% For a list of all data division functions type: help nndivide
net.divideFcn = 'dividerand';    % Divide data randomly
net.divideMode = 'sample';    % Divide up every sample
net.divideParam.trainRatio = 70/100;
net.divideParam.valRatio = 15/100;
net.divideParam.testRatio = 15/100;
% Choose a Performance Function
% For a list of all performance functions type: help nnperformance
net.performFcn = 'mse';    % Mean Squared Error
% Choose Plot Functions
% For a list of all plot functions type: help nnplot
net.plotFcns = {'plotperform','plottrainstate','ploterrhist', ...
    'plotregression', 'plotfit'};
% Train the Network
[net,tr] = train(net,x,t);
% Test the Network
y = net(x);
e = gsubtract(t,y);
performance = perform(net,t,y)
% Recalculate Training, Validation and Test Performance
trainTargets = t .* tr.trainMask{1};
valTargets = t .* tr.valMask{1};
testTargets = t .* tr.testMask{1};
trainPerformance = perform(net,trainTargets,y)
valPerformance = perform(net,valTargets,y)
testPerformance = perform(net,testTargets,y)
% View the Network
view(net)
% Plots
% Uncomment these lines to enable various plots.
```

```
%figure, plotperform(tr)
%figure, plottrainstate(tr)
%figure, ploterrhist(e)
%figure, plotregression(t,y)
%figure, plotfit(net,x,t)
% Deployment
% Change the (false) values to (true) to enable the following code blocks.
% See the help for each generation function for more information.
if (false)
    % Generate MATLAB function for neural network for application
    % deployment in MATLAB scripts or with MATLAB Compiler and Builder
    % tools, or simply to examine the calculations your trained neural
    % network performs.
    genFunction(net,'myNeuralNetworkFunction');
    y = myNeuralNetworkFunction(x);
end
if (false)
    % Generate a matrix-only MATLAB function for neural network code
    % generation with MATLAB Coder tools.
    genFunction(net,'myNeuralNetworkFunction','MatrixOnly','yes');
    y = myNeuralNetworkFunction(x);
end
if (false)
    % Generate a Simulink diagram for simulation or deployment with.
    % Simulink Coder tools.
    gensim(net);
end
sim(net,z')
```

S6 Supplementary Figures and Tables

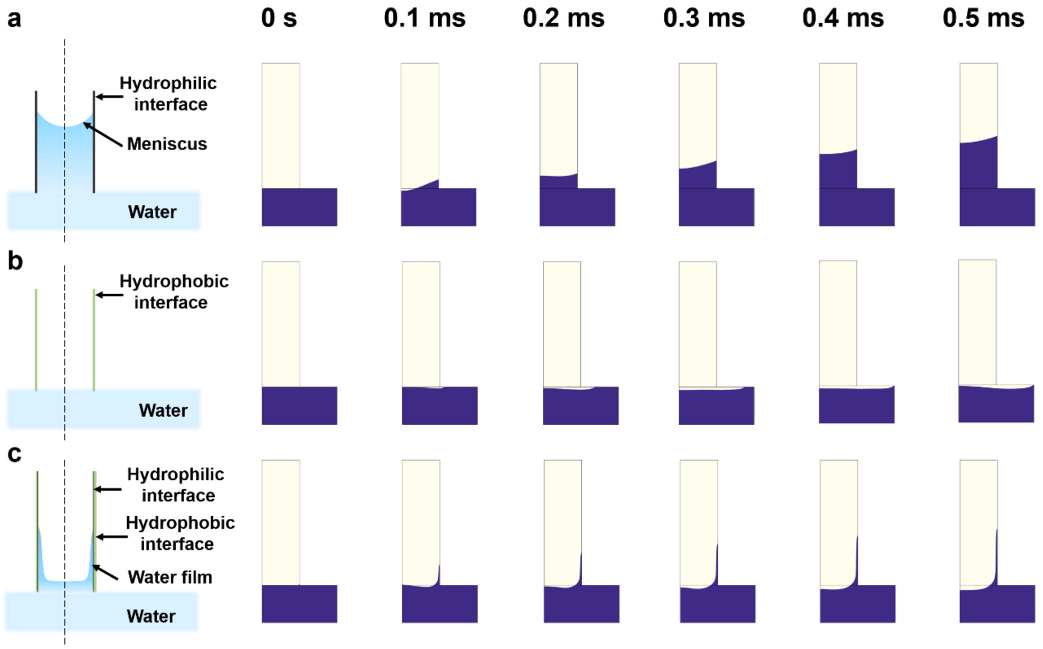

Fig. S1 The schematic and simulated results of the water transfer in the capillary with different interfaces: **(a)** The hydrophilic interface; **(b)** The hydrophobic interface; **(c)** The hydrophobic interface with a thin hydrophilic layer

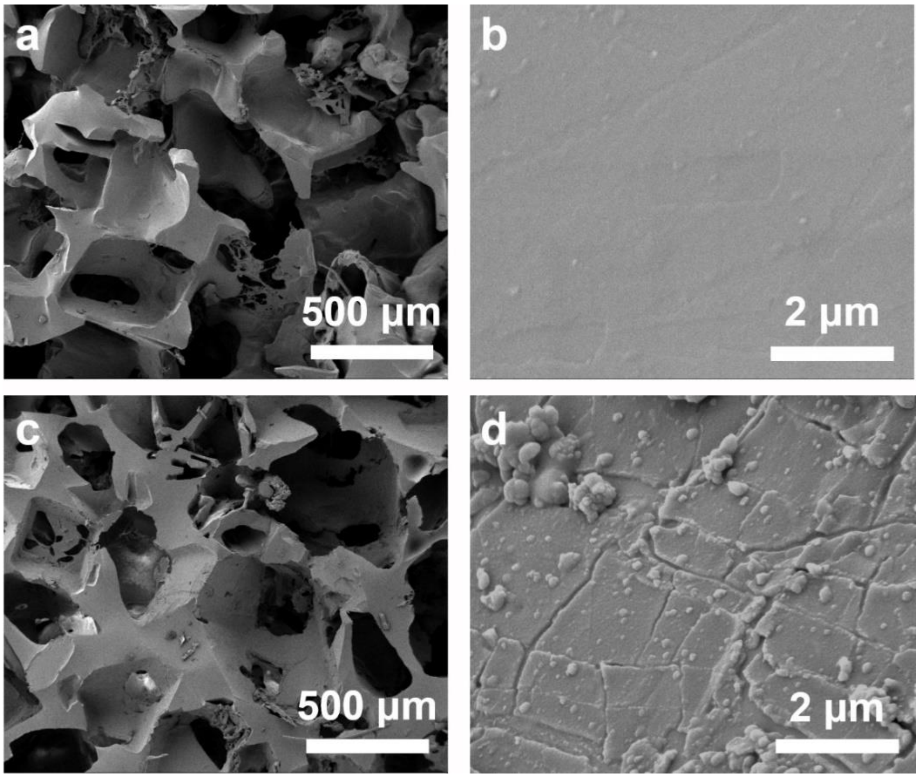

Fig. S2 SEM images of **(a)** PDMS sponge and **(b)** fiber in PDMS sponge; **(c)** PDA sponge and **(d)** fiber in PDA sponge

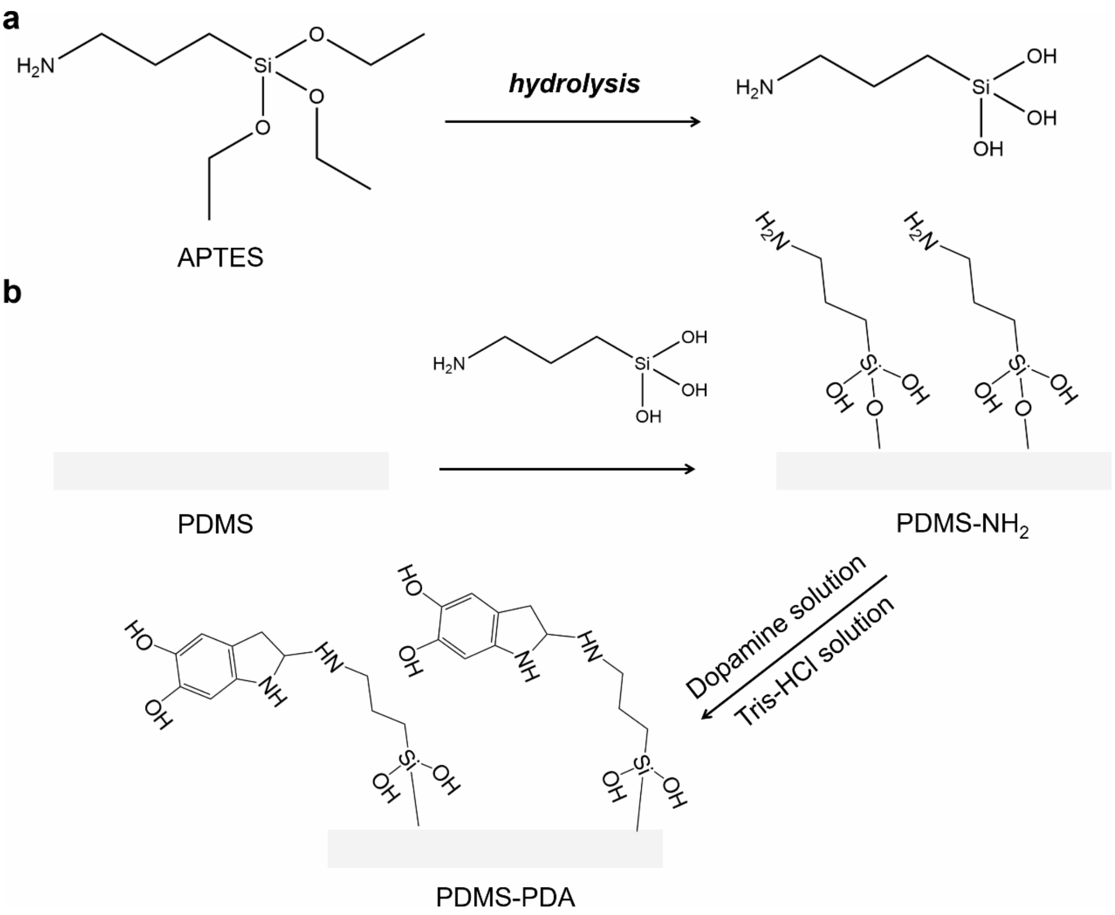

Fig. S3 The formation process of PDA on the surface of the PDMS surface [S3, S4] **(a)** The hydrolysis of APTES; **(b)** The formation of PDA on PDMS surface

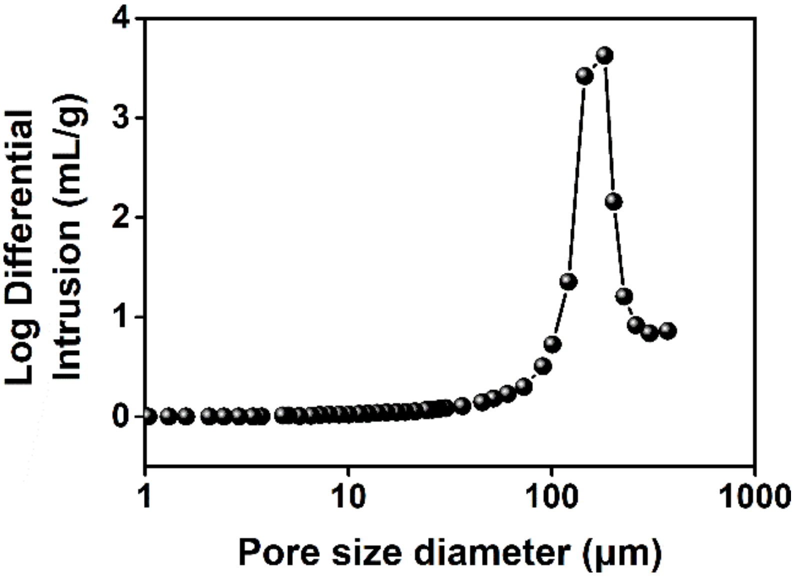

Fig. S4 Pore size distribution of PPy sponge

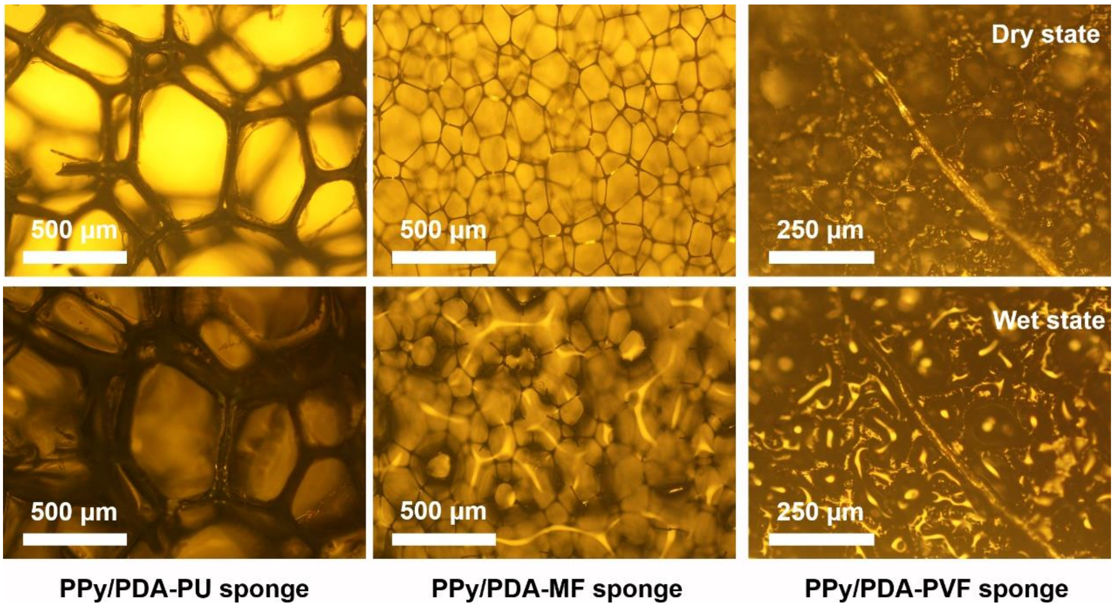

Fig. S5 The optical micrograph of the PPy/PDA-PU sponge, PPy/PDA-MF sponge and PPy/PDA-PVF sponge before and after adsorbing water

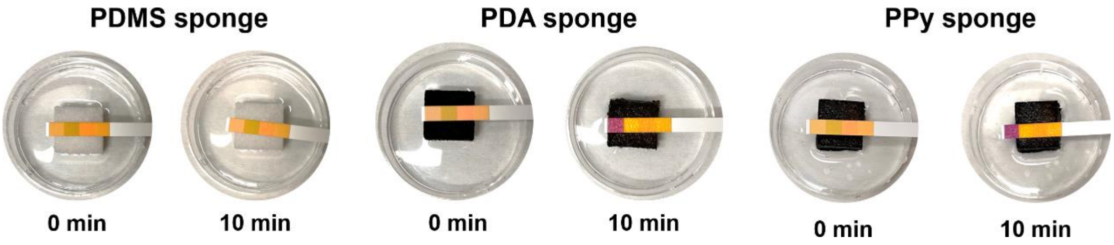

Fig. S6 The digital photos of the water transfer process in the PDMS sponge, PDA sponge, and PPy sponge. After 10 min, the pH test paper on the top of the PDA sponge and PPy sponge was wetted, while the pH test paper on the PDMS sponge was still dry. The above results implied the weak water transfer ability of the PDMS sponge

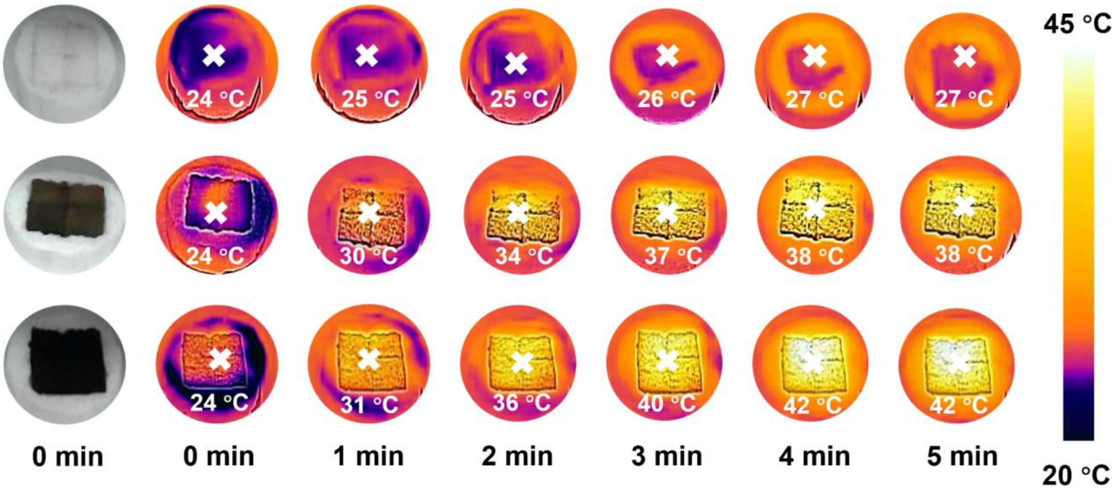

Fig. S7 The digital photos and IR photos of the PDMS sponge, PDA sponge, and PPy sponge under 1 sun

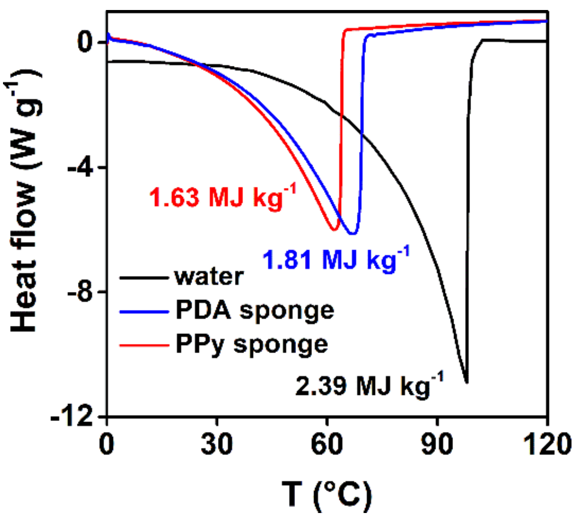

Fig. S8 DSC curves of water and water in the PDA sponge and PPy sponge

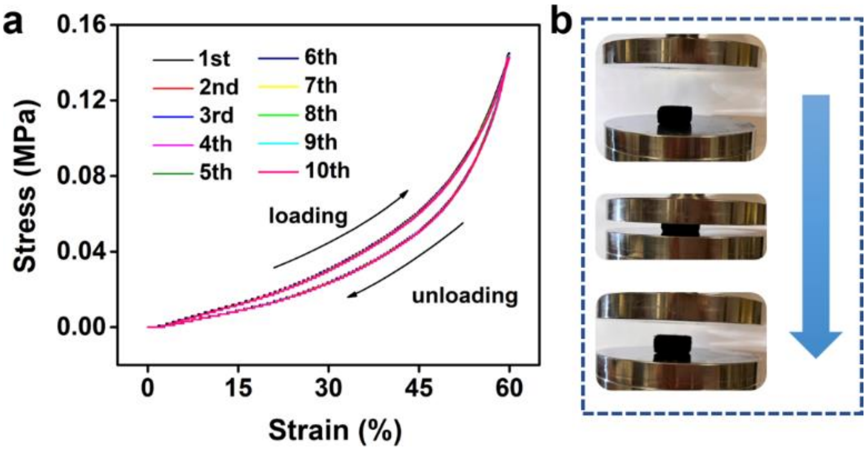

Fig. S9 (a) Cyclic stress-strain curves of PPy sponge; **(b)**The digital photos of the PPy sponge in the compress-release process

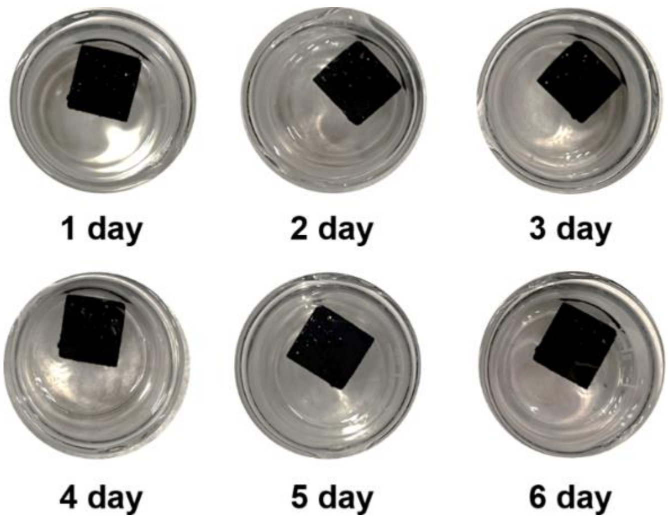

Fig. S10 The digital photos of the PPy sponge in the saturated NaCl solution

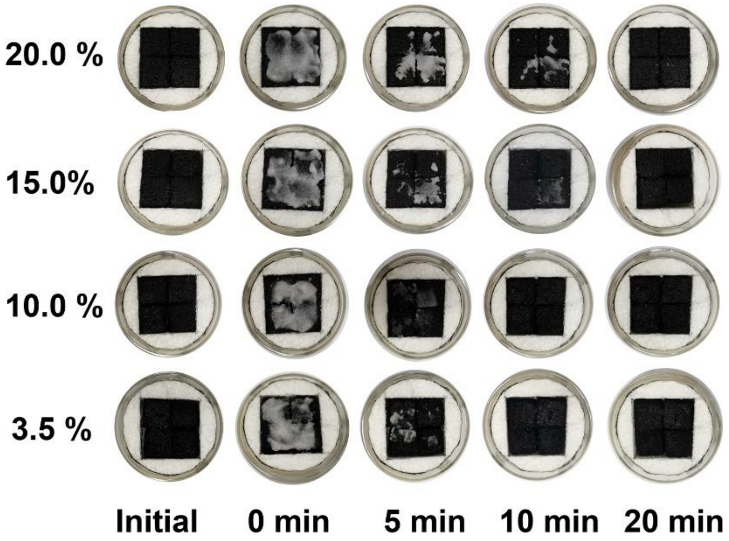

Fig. S11 The digital photos of the salt-dissolution process on the PPy sponge. 2 g of salt were sprinkled on the surface of the PPy sponge working in the different-salinity brine. After 20 minutes, the salt completely disappeared, which indicated that the PPy sponge had good salt transfer performance

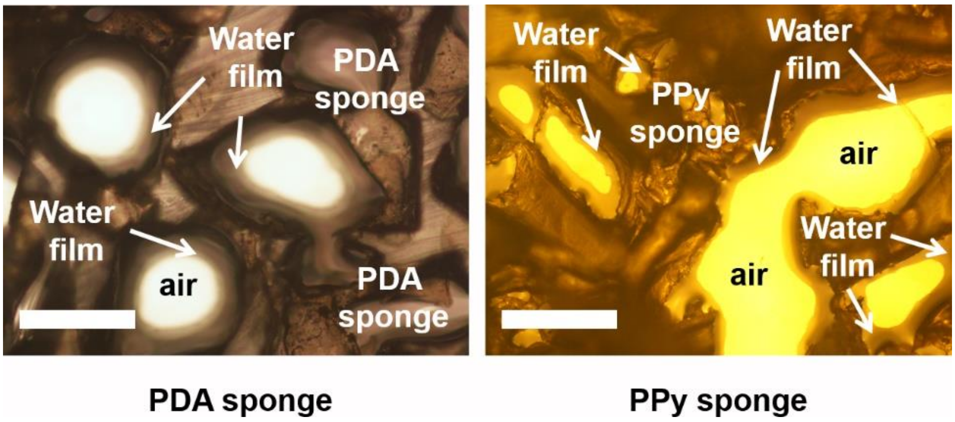

Fig. S12 The optical micrograph of the PDA sponge and PPy sponge after adsorbing water. The legend size is 200 μm

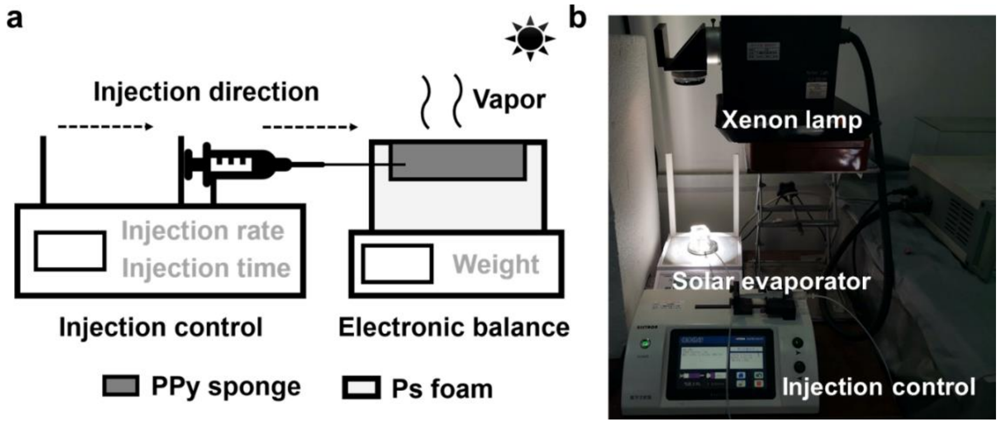

Fig. S13 (a) The schematic diagram and **(b)** digital photos of the homemade system for the in-situ water supply-evaporation experiment

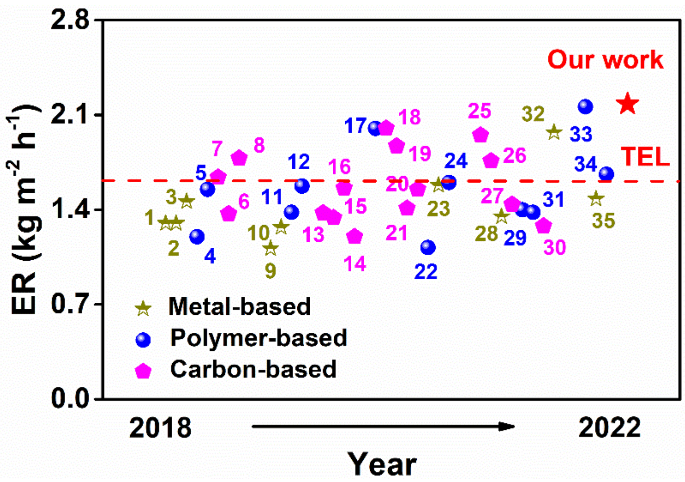

Fig. S14 The evaporation performance comparison of the reported 2D-interfacial solar evaporators and our work under 1 sun. 1-Adv. Funct. Mater. 2018, 28, 1704505; 2-ACS Appl. Mater. Interfaces. 2018, 10, 24583-24589; 3-J. Mater. Chem. A, 2018, 6, 16220–16227; 4-APPL THERM ENG. 2018, 141, 406-412; 5-ACS Sustainable Chem. Eng. 2018, 6, 10789-10797; 6-ACS Appl. Mater. Interfaces 2018, 10, 15602−15608; 7-Adv. Energy Mater. 2019, 9, 1802158; 8-Nano Energy, 2018, 46, 415-422; 9-Chem. Eng. J. 2019, 361, 999-1006; 10-J. Mater. Chem. A, 2019, 7, 11177–11185; 11-Adv. Mater. 2019, 31, 180771; 12-Nano Energy. 2019, 60, 841-849; 13-ACS Appl. Mater. Interfaces. 2019, 11, 10672−10679; 14-ACS Appl. Mater. Interfaces 2019, 11, 9974-9983; 15-Carbon, 2019, 150, 233-243; 16-Carbon, 2019, 142, 13-19; 17-Nano Lett. 2020, 20, 6051-6058; 18-Nano Energy, 2020, 78, 105322; 19-Small 2020, 16, 2000573; 20-Nano Energy, 2020, 74, 104875; 21-Chem. Eng. J. 2020, 384, 123379; 22-Adv. Mater. 2020, 32, 2004401; 23-ACS Appl. Mater. Interfaces 2020, 12, 47029-47037; 24-Cell Rep. Phys. Sci. 2020, 1, 100074; 25-Adv. Funct. Mater. 2021, 31, 2007648; 26-ACS Appl. Mater. Interfaces 2021, 13, 18829-18837; 27-Sep. Purif. Technol. 2021, 254, 117615; 28-Desalination, 2021, 511, 115116; 29-Chem. Eng. J. 2021, 417, 128051; 30-ACS Appl. Mater. Interfaces 2021, 13, 40531−40542; 31-ENERG CONVERS MANAGE. 2021, 241, 114306; 32-J. Mater. Chem. A, 2021, 9, 16805–16813; 33-Adv. Funct. Mater. 2022, 32, 2108586; 34-Carbon, 2022, 188, 265-275; 35-Chem. Eng. J. 2022, 429, 132539

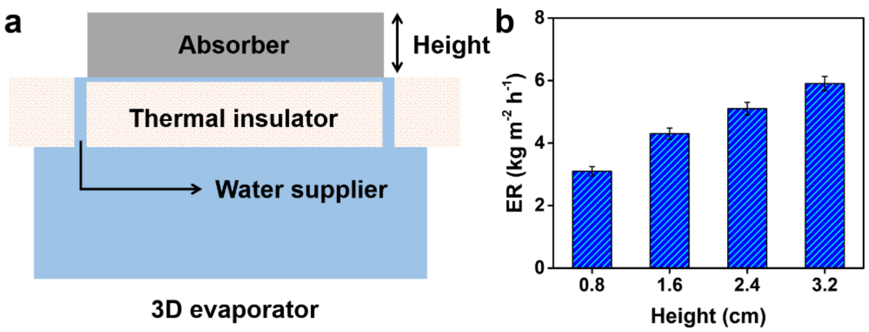

Fig. S15 (**a**) The schematic diagram of the 3D interfacial solar evaporators containing the PPy sponge; (**b**) The evaporation rate of the 3D interfacial solar evaporators containing the PPy sponge with different height

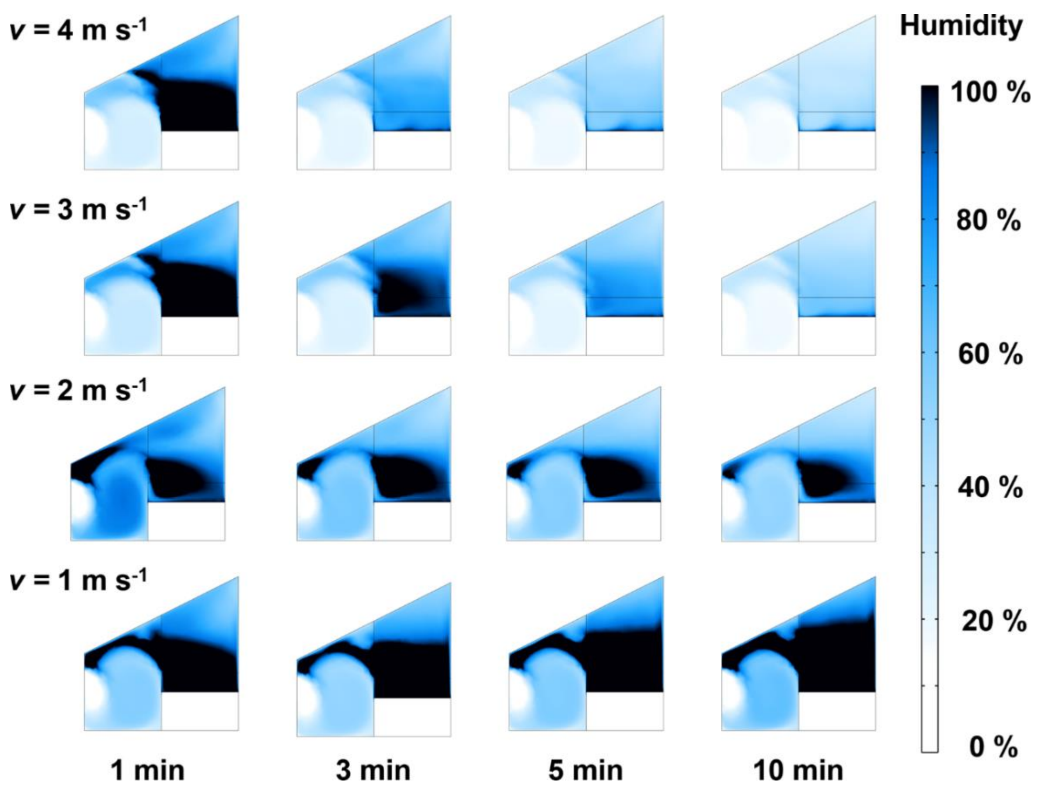

Fig. S16 The humidity field of the homemade device simulated by COMSOL software under different ventilation rates

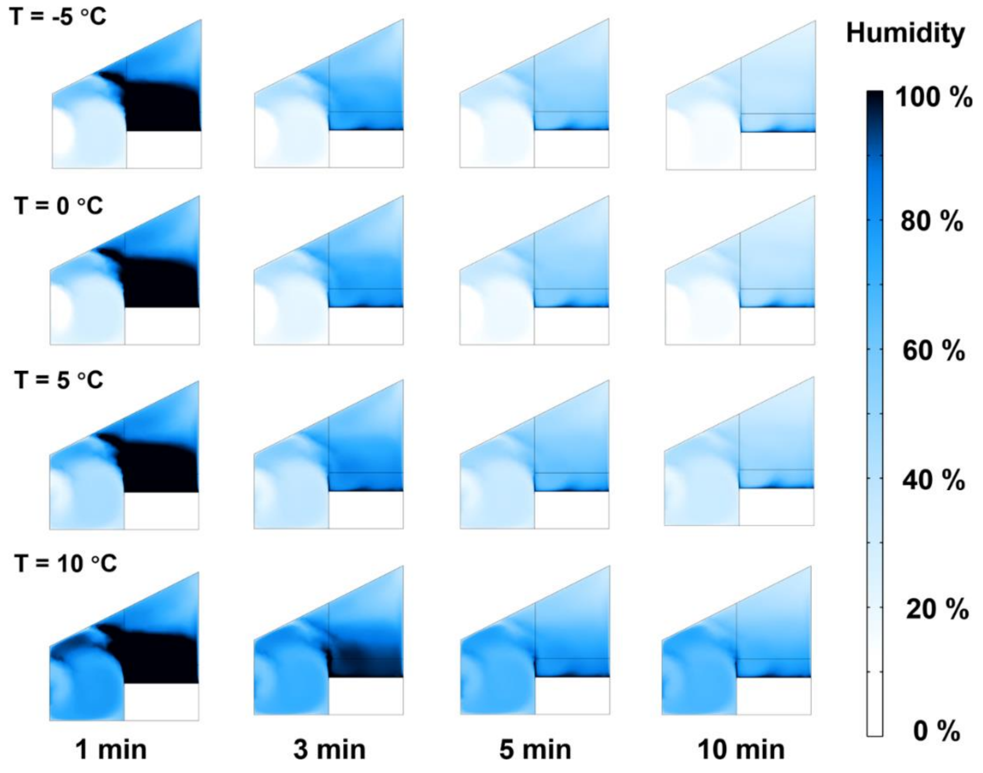

Fig. S17 The humidity field of the homemade device simulated by COMSOL software under different condensing temperatures

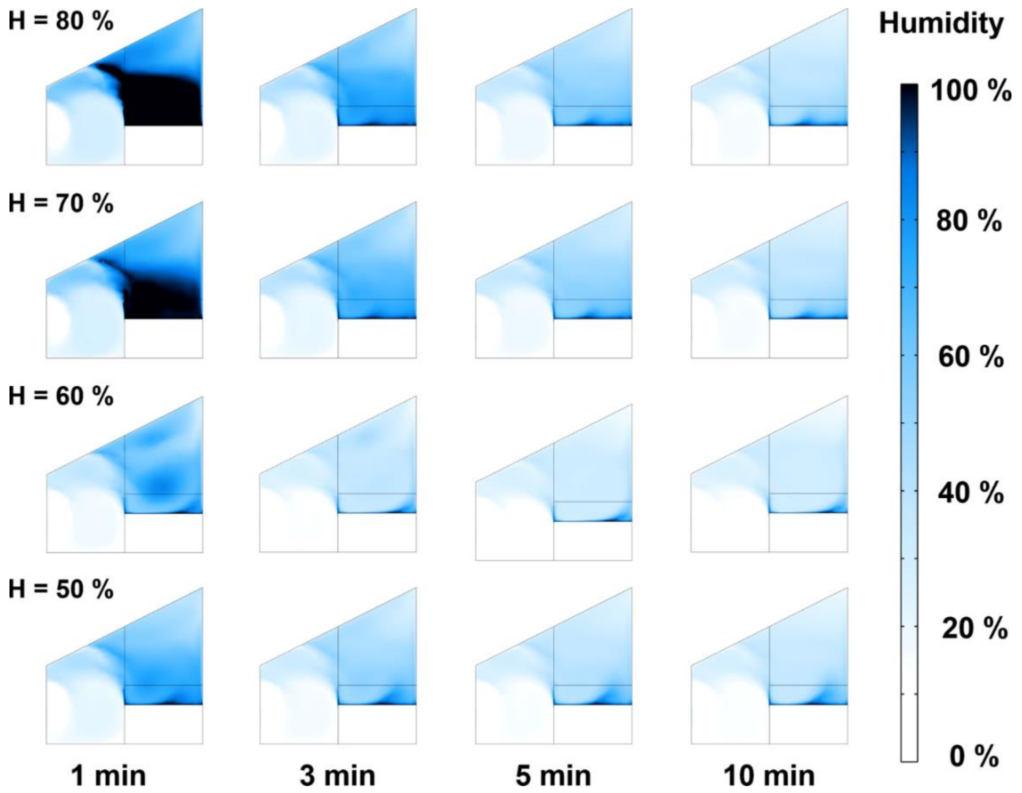

Fig. S18 The humidity field of the homemade device simulated by COMSOL software under different initial humidity

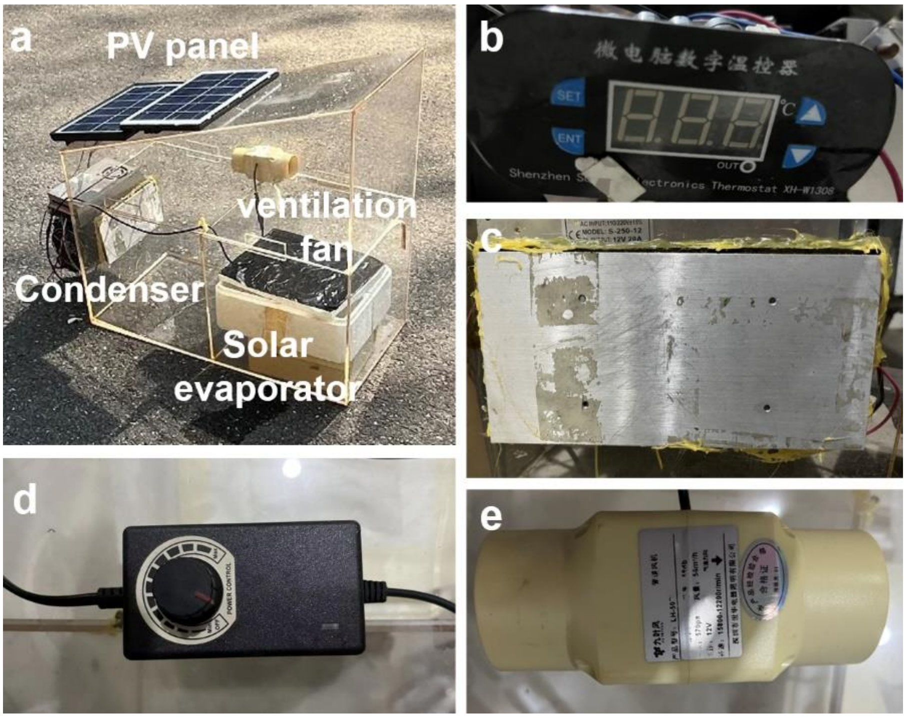

Fig. S19 (a) The digital photo of the homemade device; **(b)** the controller of the condenser; **(c)** the condenser; **(d)** the controller of the ventilation fan; **(e)** the ventilation fan

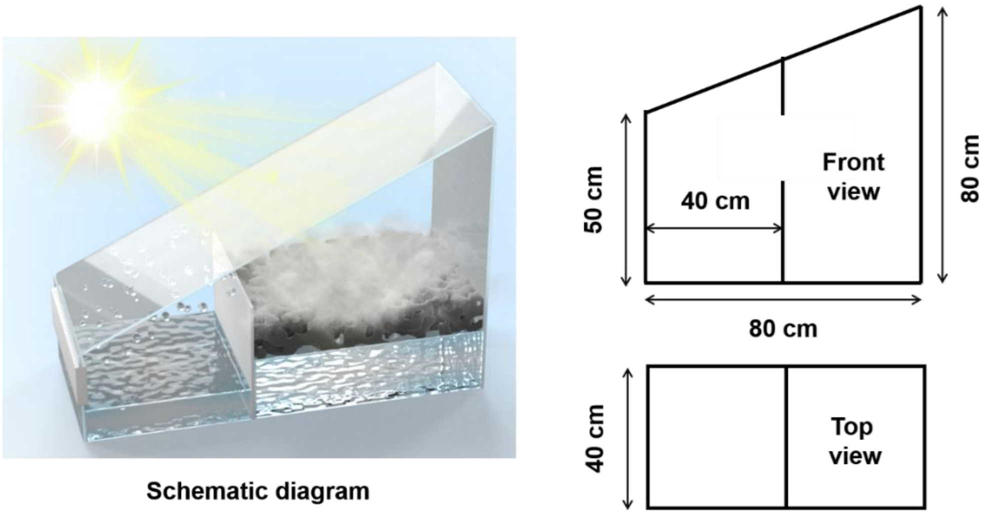

Fig. S20 The size of the homemade outdoor device. The area of the condensation chamber is same as the evaporation chamber

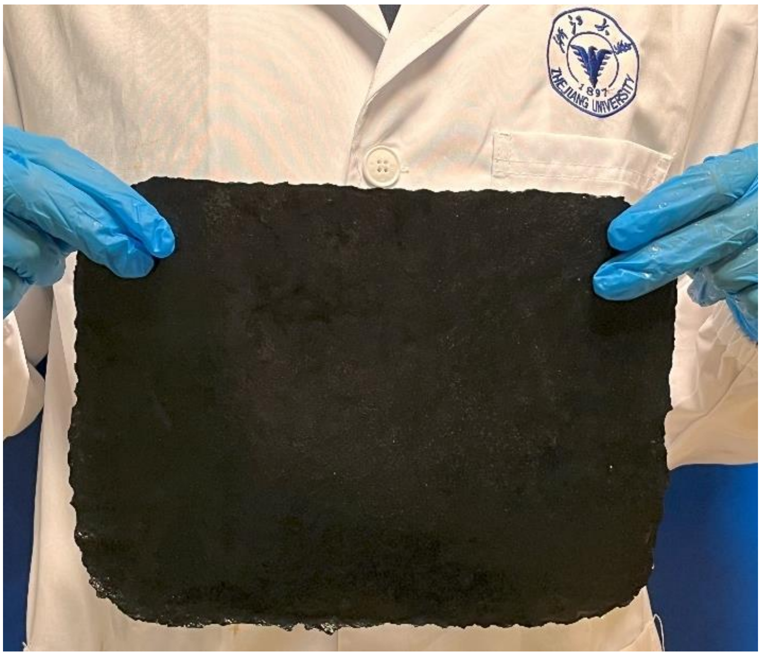

Fig. S21 The digital photos of the large-size PPy sponge

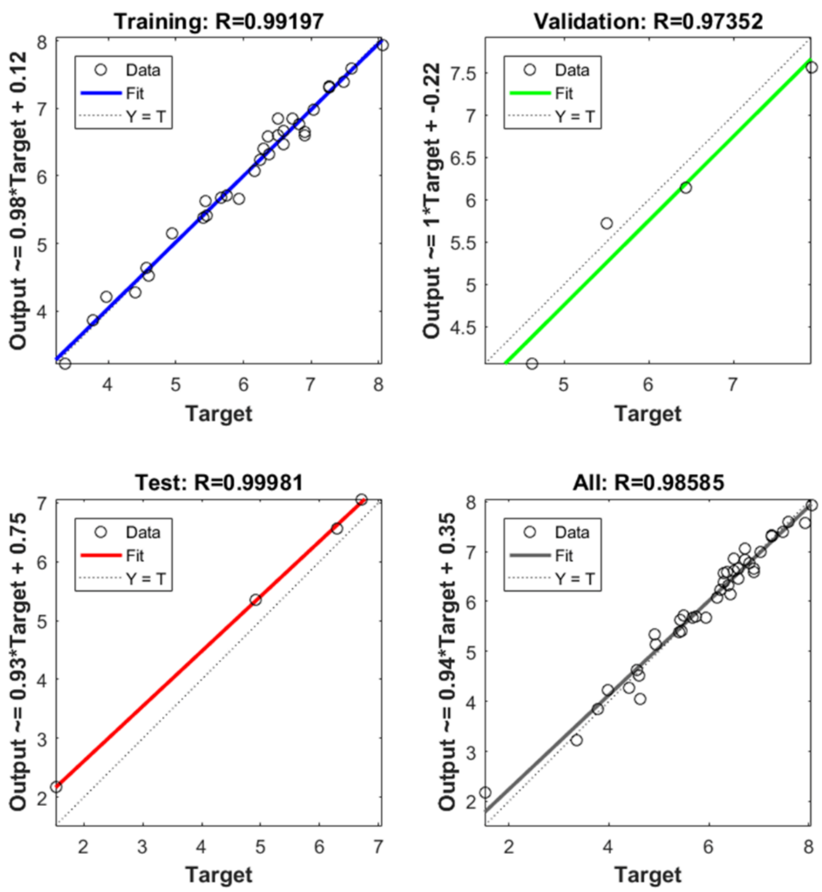

Fig. S22 Training results of the outdoor water production performance

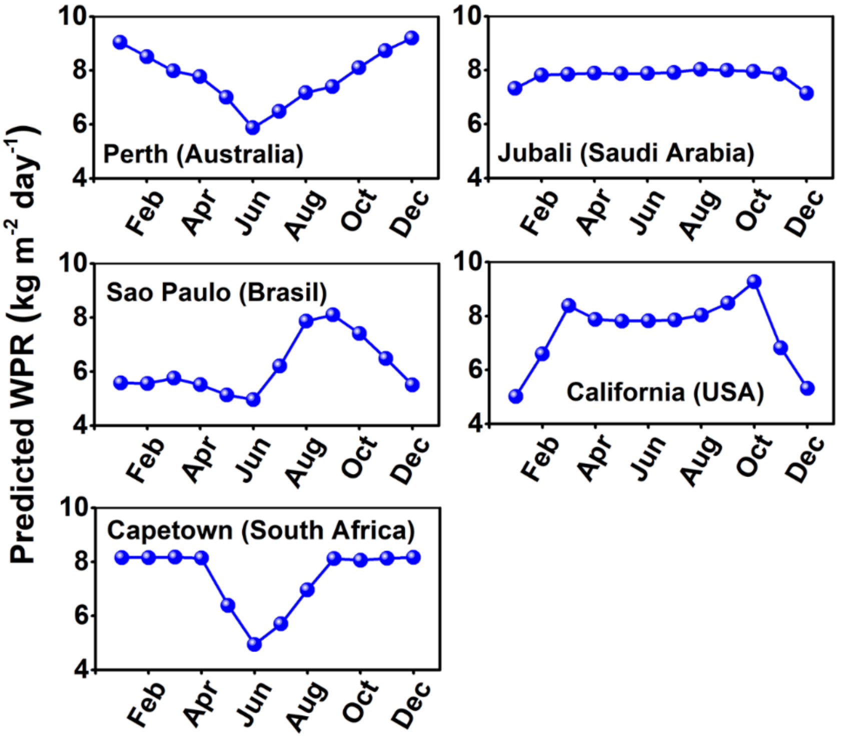

Fig. S23 The predicted outdoor water production rate in 5 water-scarce regions all over the world annually

Table S1 The TOC, COD, and TDS value of the original seawater and condensed water

	**Original seawater**	**Condensed water**
**TOC**	128.5 ± 0.2 mg L^{-1}	0.8 ± 0.1 mg L^{-1}
**COD**	385.2 ± 0.4 mg L^{-1}	2.1 ± 0.3 mg L^{-1}
**TDS**	34725.0 ± 2.0 mg L^{-1}	3.3 ± 0.8 mg L^{-1}

Table S2 The evaporation rate of the interfacial solar evaporator based on different PPy and PDA coated sponges under 1 sun

**PPy and PDA coated sponges**	**Evaporation rate (kg m^{-2} h^{-1})**
PPy/PDA-PU sponge	1.21 ± 0.05
PPy/PDA-MF sponge	1.27 ± 0.04
PPy/PDA-PVF sponge	1.23 ± 0.05

Table S3 The water production rate (kg m^{-2}) of the device under different operation mode

	**1st day**	**2nd day**
**Both the condenser and ventilation fan are off (The traditional device)**	~ 2.8	~ 1.7
**The condenser is on and ventilation fan is off**	~ 2.9	~ 1.7
**The condenser is off and ventilation fan is on**	~ 3.5	~ 2.7
**Both the condenser and ventilation fan are on (The normal operation mode of our device)**	~ 6.8	~ 5.1

Table S4 Weather conditions when measuring the water production rate of different samples

	1st day	2nd day
SF (kW h m^{-2})	7.6 ~ 7.8	7.7 ~ 7.8
T (°C)	37.7 ~ 38.2	38.0 ~ 38.4
H (%)	50 ~ 56	55 ~ 59

Table S5 The water production rate (kg m^{-2}) of the different samples

	1st day	2nd day
PPy/PDA-PVF sponge	~ 10.1	~ 9.8
PPy/PDA-MF sponge	~ 9.6	~ 9.9
PPy/PDA-PU sponge	~ 9.2	~ 9.4
PPy sponge	~ 15.6	~ 15.8

Table S6 Weather conditions when operating the device in different operating modes

	1st day	2nd day
**SF (kW h m^{-2})**	5.17 ~ 5.26	2.1 ~ 2.2
**T (℃)**	17.1 ~ 17.3	14 ~ 14.2
**H (%)**	69 ~ 72	55 ~ 59

Table S7 The weather conditions for 7 cities we selected

	Tianjin (China)			Perth (Australia)		
**Month**	**T (℃)**	**H (%)**	**Q (kW h m^{-2}h)**	**T (℃)**	**H (%)**	**Q (kW h m^{-2}h)**
**Jan.**	-3.2	57.1	2.81	23.9	54.1	8.45
**Feb.**	0.4	55.2	3.71	24	54.3	7.53
**Mar.**	7.1	52.4	4.75	22.4	57.8	6.02
**Apr.**	14.7	53.7	5.78	19.4	64.1	4.38
**May.**	20.3	58.9	6.26	16.2	71.5	3.16
**Jun.**	24.5	68.5	5.76	13.5	76.1	2.6
**Jul.**	26.7	78.3	5.12	12.8	76.4	2.87
**Aug.**	25.8	78.5	4.76	13.3	74.1	3.75
**Sept.**	21.2	70.6	4.43	14.7	70.9	5.1
**Oct.**	14	66.1	3.72	16.6	64.6	6.56
**Nov.**	5.3	64.7	2.82	19.6	59.5	7.75
**Dec.**	-1.2	61.1	2.47	22	55.8	8.5
	**Sao Paulo (Brasil)**			**California (USA)**		
**Month**	**T (℃)**	**H (%)**	**Q (kW h m^{-2}h)**	**T (℃)**	**H (%)**	**Q (kW h m^{-2}h)**
**Jan.**	25.6	90.5	4.5	5.4	68	2.5
**Feb.**	25.6	90	4.44	6	71.7	3.49
**Mar.**	25.7	89.8	4.54	0.2	69.9	4.88
**Apr.**	25.6	90.4	4.45	11.1	62	6.21
**May.**	25.4	90.3	4.2	15.7	50.4	7.32
**Jun.**	25.1	89.1	4.01	20.8	35.7	8.18
**Jul.**	25.1	87.2	4.4	25	26.9	8.15
**Aug.**	25.8	85	4.92	24.2	26	7.36
**Sept.**	26.1	85.2	5.09	20.9	30.6	5.96
**Oct.**	26.2	86.3	4.93	15.3	39.7	4.43
**Nov.**	25.9	88.9	4.81	9.3	53.5	2.89
**Dec.**	25.7	90.3	4.45	5.3	63.2	2.31
	**Capetown (South Africa)**			**Jubali (Saudi Arabia)**		
**Month**	**T (℃)**	**H (%)**	**Q (kW h m^{-2}h)**	**T (℃)**	**H (%)**	**Q (kW h m^{-2}h)**
**Jan.**	20.4	68	7.72	17.2	65.2	3.53889
**Feb.**	20.4	69.9	7.05	17.5	65.1	4.48056
**Mar.**	19.2	72.6	5.86	19.7	63.4	5.16111
**Apr.**	16.9	76.6	4.17	23.8	60.3	5.83889
**May.**	14.4	79.6	2.97	28.6	54.2	6.86944

**Jun.**	12.5	79.9	2.45	31.9	51.1	7.7
**Jul.**	11.9	78.9	2.62	33.8	52.7	7.35
**Aug.**	12.4	78.6	3.4	34.2	56.5	7.03889
**Sept.**	13.7	76.6	4.75	32.3	57.2	6.45
**Oct.**	15.6	71.6	6.09	29.3	58.1	5.26111
**Nov.**	17.9	68.9	7.48	24.5	60.1	3.93056
**Dec.**	19.5	68.4	7.85	19.7	64.7	3.28889
	**Singapore**					
**Month**	**T (°C)**	**H (%)**	**Q (kW h m^{-2}h)**			
**Jan.**	25.8	84.5	4.63056			
**Feb.**	26.4	82	5.1			
**Mar.**	26.8	83	4.96944			
**Apr.**	27.2	83.8	4.61944			
**May.**	27.5	83.3	4.35			
**Jun.**	27.4	81.8	4.3			
**Jul.**	27.1	82	4.35			
**Aug.**	27	82	4.41944			
**Sept.**	26.8	82.6	4.51944			
**Oct.**	26.8	82.9	4.33889			
**Nov.**	26.3	85.5	3.91111			
**Dec.**	25.7	86.5	3.9			

Supplementary References

[S1] Z. Yu, S. Cheng, C. Li, L. Li, J. Yang, Highly efficient solar vapor generator enabled by a 3D hierarchical structure constructed with hydrophilic carbon felt for desalination and wastewater treatment. ACS Appl Mater Inter. **11**(35), 32038-32045 (2019). https://doi.org/10.1021/acsami.9b08480

[S2] J. Xu, Z. Wang, C. Chang, B. Fu, P. Tao et al., Solar-driven interfacial desalination for simultaneous freshwater and salt generation. Desalination. **484**, 114423 (2020). https://doi.org/https://doi.org/10.1016/j.desal.2020.114423

[S3] F. Bernsmann, V. Ball, F. Addiego, A. Ponche, M. Michel et al., Ruch. Dopamine-melanin film deposition depends on the used oxidant and buffer solution. Langmuir. 27(6), 2819-2825 (2011). https://doi.org/10.1021/la104981s

[S4] Y. Liu, K. Ai, L. Lu, Polydopamine and its derivative materials: Synthesis and promising applications in energy, environmental, and biomedical fields. Chem Rev. **114**(9), 5057-5115 (2014). https://doi.org/10.1021/cr400407a
